# Determination of Cd, Pb, and Cu in the Atmospheric Aerosol of Central East Antarctica at Dome C (Concordia Station)

**DOI:** 10.3390/molecules26071997

**Published:** 2021-04-01

**Authors:** Silvia Illuminati, Anna Annibaldi, Cristina Truzzi, Caterina Mantini, Eleonora Conca, Mery Malandrino, Giada Giglione, Matteo Fanelli, Giuseppe Scarponi

**Affiliations:** 1 Dipartimento di Scienze della Vita e dell’Ambiente, Università Politecnica delle Marche, Via Brecce Bianche, 60131 Ancona, Italy; s.illuminati@univpm.it (S.I.); caterina.mantini@libero.it (C.M.); g.giglione@pm.univpm.it (G.G.); matteo.fanelli@virgilio.it (M.F.); g.scarponi@univpm.it (G.S.); 2 Department of Analytical Chemistry, University of Torino, Via Giuria 5, 10125 Torino, Italy; eleonora.conca@unito.it (E.C.); mery.malandrino@unito.it (M.M.)

**Keywords:** cadmium, lead, copper, atmospheric aerosol, Central East Antarctica, Dome C, Concordia Station, background contents, local contamination

## Abstract

Trace heavy metals Cd, Pb, and Cu were determined (by square wave anodic stripping voltammetry) in aerosol samples collected at Dome C (the Italo-French Station Concordia), a remote site of the Central East Antarctic plateau, for which no data are available until now. During the Austral Summer 2005–2006, three PM10 high-volume impactors were installed in two locations nearby of Concordia station: the first one very close and downwind of the station (about 50 m north), the other two (very close to each other) in a ‘distant’ site, upwind of the station and close to the astrophysics tent (not used in that expedition) at ~800 m south of Station Concordia. For each sample, the availability of the mass of the aerosol collected (obtained by differential weighing carried out on site), in addition to the volume of the filtered air, allowed us to express results both in terms of metal mass fractions in the aerosol and in the usual way of metal atmospheric concentrations. Metal contents increased in the order Cd < Pb < Cu with the following ranges of values: Cd 1.0–8.4 µg g^−1^ (0.09–3.1 pg m^−3^), Pb 96–470 µg g^−1^ (12–62 pg m^−3^), and Cu 0.17–20 mg g^−1^ (0.027–2.4 ng m^−3^). From the metal temporal profiles obtained we estimated the following background values for the area of Dome C, expressed both in mass fractions and in atmospheric concentrations: Cd 1.2 ± 0.2 µg g^−1^ (0.24 ± 0.13 pg m^−3^), Pb (here fixed as upper limit) 113 ± 13 µg g^−1^ (21 ± 8 pg m^−3^), and Cu 0.91 ± 0.48 mg g^−1^ (0.12 ± 0.07 ng m^−3^). The highest values were observed in the first part of the season, and particularly for the site close to the station, possibly related to sample contamination linked to intense activity at the Concordia station connected with the beginning of the expedition, including aircraft arrivals/departures. Increments of up to 10 times (and even 20 times for Cu) were recorded with respect to the background values. The metal excesses of the contaminated over background samples were found approximately, except for Cu, in the same proportion of the metal contents of the special Antarctic blend (SAB) diesel fuel, which is used almost exclusively at Concordia Station. The effect of the wind direction was also observed. Thus in the intermediate period of the campaign, when the wind direction reversed for several days with respect to the prevailing one, Cd and Pb metal contents decreased at the sampling point installed close to the station, now upwind of Concordia station, and increased at the ‘clean’ site astrophysics tent, turned downwind at the main station. No simple and easily interpretable effect of the wind direction was observed for Cu, which suggests that some other extemporaneous and not clearly identified factor may have intervened in this case. These results suggest that the human impact at Dome C influences mainly the zone very close to the station, but also the area in the neighborhood, including the supposed clean site of the astrophysics tent (about 800 m far from the station), when the wind direction reverses with respect to the prevailing one, leaving the site downwind of the station Concordia. Since no other data are reported for the Dome C area, our results are compared with literature data referred to the South Pole Station (the only other plateau site for which data are available) and several other coastal Antarctic sites, observing that our results (excluding Cu) are the lowest ever observed for Antarctic aerosol.

## 1. Introduction

Antarctica is the most remote continent in the world, and thus it is characterized by the lowest direct human pressure than any other [1]. Really, it is considered an excellent natural laboratory where to study the natural bio-geochemical processes of major and trace elements (and other chemicals in general), and then to better understand the impact of human activity both from local and remote sources, the latter from long-range transport of aerosol from other continents. This opportunity makes it possible to contribute significantly to the research on past and present global changes, both climatic and environmental in general [2]. Moreover, knowledge of the chemical composition of the atmospheric aerosol in the air, snow and ice of the Antarctic continent, is an essential pre-requisite for the interpretation of data obtained from ice cores aimed at reconstructing the changes that occurred in the terrestrial atmosphere in the past and at estimating the climatic and environmental changes expected for the future [3,4,5,6,7,8,9,10,11,12].

Much research on aerosol chemistry and physics in Antarctica deals with coastal sites—e.g., stations Halley, Terra Nova Bay, Dumont d’Urville, Aboa, Maitri, Neumayer, and Antarctic Peninsula [13,14,15,16,17,18,19,20,21,22,23,24,25,26]. Far fewer studies refer to inland areas (see Section 2.3. for a review of literature data referring to the metals of interest here), because of the extreme meteo-climatic conditions existing in Central Antarctica and the difficulties in the analytical procedures concerning the management of extremely pure samples. Indeed, the available studies show that aerosol has much lower atmospheric concentration in inland sites than in coastal areas. This fact makes the application of the direct gravimetric method for the determination of aerosol mass (and then its atmospheric concentration) highly challenging for Central Antarctica. In fact, initially it was just used for coastal sites [14,19,22,27,28,29,30]. Only indirect methods were applied to estimate the aerosol mass in inland sites of South Pole [31,32,33,34,35,36,37], East Antarctic Plateau [38], and Dome C [39,40,41], until recently when the direct differential gravimetry method was used by us for the first time on a plateau site, at Dome C [42]. PM10 aerosol atmospheric concentrations were reported with reference to both standard air volume (298 K, 760 mmHg) and actual air volume (more used in the literature).The observed values were the lowest ever reported for Antarctica: range 0.089–0.455 µg m^−3^, mean value 0.22 µg m^−3^, expressed at the “actual conditions” of temperature and pressure at sampling site. These units instead of standard air were chosen often in order to reconstruct the real load of aerosol components in the local atmosphere [40].

These studies have shown that Central Antarctica shows the lowest mass concentrations of atmospheric aerosol on the Earth. Therefore, it can be considered the best place to study the background aerosol composition [43].

The object of the present study refers to the determination of trace metals cadmium, lead, and copper in the same samples collected at Dome C for which aerosol atmospheric concentrations has already been reported [42]. The choice of these elements is linked to several factors. Typically, their presence in the Antarctic aerosol is associated to anthropogenic emissions [44,45,46,47]. Indeed, high enrichment factors with respect to mean crustal and seawater compositions have usually been attributed to inputs from human activities either local in Antarctica and/or remote in the Southern Hemisphere by long-range transport [48,49,50]. The same elements are among those released by the combustion of coal, oil, and gasoline and by non-ferrous metal production [51]. Cadmium and lead are considered 2 of the 10 chemicals of major health concern by the World Health Organization, with a wide range of toxicity and long elimination half-life [52]. Finally, but not less important, Cd, Pb, and Cu at ultratrace levels in the aerosol can be analyzed simultaneously without too much difficulty by voltammetry [53].

Cadmium is a high toxicity chemical element, present at trace/ultratrace levels in the Earth’s crust. It tends to be concentrated in sulfide, especially rich in zinc minerals [46,54,55]. In the last 100 years, the increased presence of cadmium in Antarctica has been attributed to anthropic emissions [46]. Indeed, Antarctic ice recorded an increasing Cd deposition in response to long-range industrial emissions [48,56,57,58]. However, the available literature also supports the hypothesis that cadmium could be associated to natural sources. In fact, the analyses of ice core from Dome C and Vostok (about 500 km far from Dome C), suggest that Cd could have had a volcanic source (note that volcanoes Erebus, Melbourne, and Rittmann are present in East Antarctica) during the cold and warm climatic ages [57,59]. It has been shown that the volcano Erebus emits, especially during the summer, a sulfide and metal rich plume with high enrichment factors for Cd (both with respect to magma and crust), and that its dispersion can reach the inland Antarctica, in particular the Central East Antarctica [60]. Moreover, it was estimated that Cd of volcanic origin contributes to about 40–50% of the total annual worldwide emissions [61].

As for lead, the low background atmospheric concentrations together with the well-known and often distinct isotopic characteristics of industrial sources, make this element an ideal tracer of industrial pollution [44,62,63,64,65,66,67,68]. Many authors have investigated about Pb as a pollutant also in Antarctica [44,64,69]. It was also shown that the total content of Pb in snow of pre-industrial times could be explained by crustal and volcanic contributions, about two-thirds and one-third, respectively [70]. Marine contributions were evaluated as negligible. Higher concentrations of Pb were found in recent Antarctic snow which could not be attributed entirely to these sources, and the only explanation for this increase was ascribed to anthropogenic pollution [71,72,73]. The continental areas implicated in long-range transport of Pb were shown to be mainly South America and/or Australia, depending on the specific site [44,74]. Recent studies of Pb in Antarctic ice/snow have shown that concentrations first showed signs of anthropogenic emissions during the late 19th century after that increased very rapidly in early 20th century [74]. Concentrations decreased during the Second World War, then rose again since ~1950s in association with increasing trends in mining and smelting of non-ferrous metal ores and in the consumption of leaded gasoline [44,64,75,76]. Such increasing emissions reached a maximum during the 1980s due to the widespread use of motor vehicles and of leaded petrol. More recent concentrations in snow and firn during the 1990s and 2000s have decreased, coinciding with the phase-out of leaded petrol [74,76,77].

Copper concentration in the atmospheric aerosol of the Earth is too high to be explained in terms of normal crustal weathering processes. It is one of the most common pollutants of the environment and the source of contamination is reported to be of anthropogenic nature [78]. Indeed, copper in Antarctica has been attributed to an anthropic origin, mainly connected to the local impact of the stations [2,14,45,47]. On the other hand, mineralogical analyses, and chemical, geochemical, paleontological, and isotopic determinations—which have been carried out on southern South American soil dusts and on the Vostok, Dome C and Dome B ice core dusts—together with model-based investigations, have demonstrated that dust deposited in the past on the East Antarctic plateau has a predominant origin from the southern South America (Patagonian desert, Tierra del Fuego) and the Antarctic Peninsula [79,80,81,82,83,84,85,86,87,88,89]. The same origin has also been proved for aerosol collected at Dome C during 2005, just the same year that the samples of the present work were taken, from which it was concluded that particulate matter produced from modern dust activity in Patagonia reach the east Antarctic plateau [90]. Thus, in regards to possible natural origins of the metals of our interest, it is important to note that copper is the second most abundant metal, after iron, in mineral occurrences and deposits in Antarctica, while lead is present only in very minor quantities, and cadmium, as far as is known, is not present at all. In particular, along the east coast of the Antarctic Peninsula there are many exposed rock outcrops containing copper ores, copper-molybdenum porphyry deposits, while in minor amounts copper was also found in metamorphic and intrusive rocks of East Antarctica and Transantarctic Mountains not covered by ice [91,92,93]. The same high copper content is present on dust from the southernmost part of South America, which together with the Antarctic Peninsula (a geological continuation of South American Andes) is part of the Andean copper province and belongs to the circum-Pacific copper belt [91,94,95,96].

Currently, only the major elements in aerosol have been determined at Dome C, from 2004 to 2013 [40,41,97]. As concerns the trace metal that are object of the present study, most data available on Antarctic aerosol mainly refer to coastal areas, and the only data dealing with inland sites are related to South Pole, starting from austral summer 1970 until 2003 (for an extensive literature reference see Section 2.3.). This is possibly due to difficulties in the treatment and analysis of samples under uncontaminated conditions and with sufficient sensitivity [4,7,98].

The experimental design was planned in order to reach two main objectives: (i) to establish for the first time background values of Cd, Pb, and Cu in the aerosol of Dome C area, determining the metal concentrations in a clean area, under the hypothesis of no effect of the station; and (ii) to investigate the impact of Concordia Station on the trace metal concentrations in the local aerosol concentration, analyzing samples collected under the direct effect of the station. This study fits into two of the six priorities set by the Scientific Committee on Antarctic Research (SCAR), in particular priority no. 1 (“Define the global reach of the Antarctic atmosphere and Southern Ocean” or “Antarctic atmosphere and global connections”), and no. 6 (“Recognize and mitigate human influences” or “Human presence in Antarctica”, under the question no. 74: “How can natural and human-induced environmental changes be distinguished, and how will this knowledge affect Antarctic governance?”) [99,100].

## 2. Results and Discussion

### 2.1. Back Trajectories and Air Masses Arriving at Dome C

For a thorough understanding of metal content temporal profiles, and then to evaluate the potential impact of local and remote sources, we need information related to the pathways of particles arriving day by day at Dome C (backward trajectories). These, in turn, are related to the local wind direction and to the general circulation of the air masses in the area of Central East Antarctica including more distant sites too.

In recent years, backward trajectories have been used more and more frequently as powerful tools to observe and classify air pathways in a simple way, to speculate about remote origins of airborne particulate matter, to provide evidence of long-range transport of aerosol particles, and to evaluate source apportionments of pollutants. As non-Antarctic examples we can quote studies carried out in Hungary [101,102]; in Northern Ireland [103]; in the three cities of Madrid, Birmingham, Athens [104]; in Zimbabwe [105]. For an extensive review of non-Antarctic literature, the reader is referred to a review paper on backward trajectories applications in interpreting observed atmospheric composition [106].

More recently, Antarctica applications have also been reported both for coastal and inland sites, including the plateau areas and Dome C in particular. A non-exhaustive selection includes papers referring to: (i) coastal sites of Queen Maud Land (Neumayer Stn), Victoria Land (Mario Zucchelli Stn), Antarctic Peninsula (King Sejong Stn, Larsen C Ice Shelf, Gabriel De Castilla Stn), and Southern Ocean (Pacific Sector); (ii) inland sites (Gawn Ice Piedmont, David Glacier, ITASE GV5 point); and (iii) plateau sites (Mid Point, Dome C, Law Dome, Talos Dome, Taylor Dome) [41,107,108,109,110,111,112,113,114,115,116,117,118].

**Figure 1:**
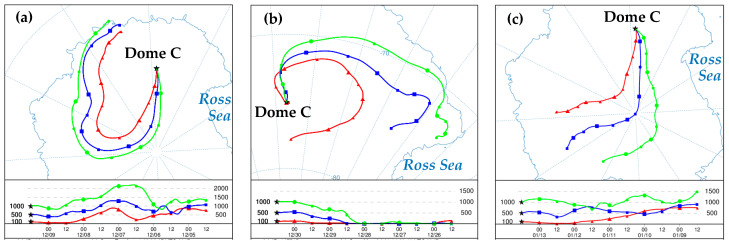
Examples of NOAA Hysplit five-day air backward trajectories run for arrival heights of 100 m (red), 500 m (blue), and 1000 m (green) a.g.l. at 12 am recorded at Dome C during the summer 2005–2006. (**a**) 9 December 2005; (**b**) 30 December 2005; (**c**) 13 January 2006.

The analysis of air masses arriving at Dome C were carried out through air back trajectories calculated for each day of the sampling period and reported in Figures S1a (7–20 December), S1b (21 December–1 January), and S1c (2–14 January) of the Supplementary Materials. Note that this time division corresponds to the sampling periods 1, 2, and 3 of sampling points CS and AT1. These trajectories allowed us to evaluate a complete scenario of air masses coming from local and remote areas revealing flying particles of different origins that could influence the sampling site. During the first sampling period (Figure S1a), the pictures suggest that the air masses originated and passed through inland of East Antarctica, coming mainly from the south, southwest during the main part of the period. Some contribution from coastal regions was also present for 9, 10 December (partly) and 17 (see the location of the particles 4–5 days before arrival). A typical situation within the first period is shown in Figure 1a (ref. 9 December 2005). During the second period (Figure S1b), air masses continued to come from South until 26 December, when they began to change direction, and from 27 December to 1 January, underwent a complete inversion of direction arriving from North instead of from South (see typical trajectories for this case in Figure 1b, 30 December 2005). From the corresponding pictures, it is clearly visible contributions of air masses coming from north–northeast, carrying out particles from marine and coastal regions. During the last part of the season—i.e., 2–14 January (Figure S1c)—the major contribution of air masses came from south, southwest, flying across inland sites of Antarctica (see for example Figure 1c, 13 January, 2006). A residual contribution from coastal sites was present in the 2 and 3 January.

These results agree well with, and further detail, previous observations on wind direction in the area during the sampling period. Thus, we will take them into account in the interpretation and discussion of data on the content of metals and their temporal profiles. In short: (i) the sampling point Concordia was mainly downwind of the station in all the sampling periods 1 and 3, while for period 2 it remained in part upwind, i.e., from 27 December, 2005, to 1 January, 2006. Exactly the reverse happened for the sampling point AT1, which was always upwind of the station in periods 1 and 3, while for period 2 it was in a downwind part of the time (27 December–1 January). For the point AT2, due to the different sampling times, it happened that in both periods 1 and 2 we had a part of time with wind direction from north instead of from south, leaving the point downwind of the station, i.e., from 27 and 28 December for the period 1, and 29 December–1 January for the period 2.

### 2.2. Metal Contents and Temporal Profiles

The present discussion takes advantage of the availability of data on total metal contents in aerosol samples, which are expressed both in terms of mass fractions and in atmospheric concentrations (the latter unit more usually reported in the literature). The first quantity represents the amount of metal present per unit mass of aerosol, while the second one is the amount of metal present per unit volume of air in the particulate form, which is related to both the metal mass fraction and the aerosol atmospheric concentration. In fact, the data available on the aerosol atmospheric concentrations [42] link the two ways of expressing metal contents. Thus, we can discuss separately our results both in metal mass fractions, to understand the aerosol chemical composition, and in metal atmospheric concentrations, which are related to the total metal amount in the air.

All data reported are intended as total contents obtained from complete digestion of samples and after blank subtraction. Atmospheric concentrations refer to standard air (298 K, 760 mmHg).

**Figure 2:**
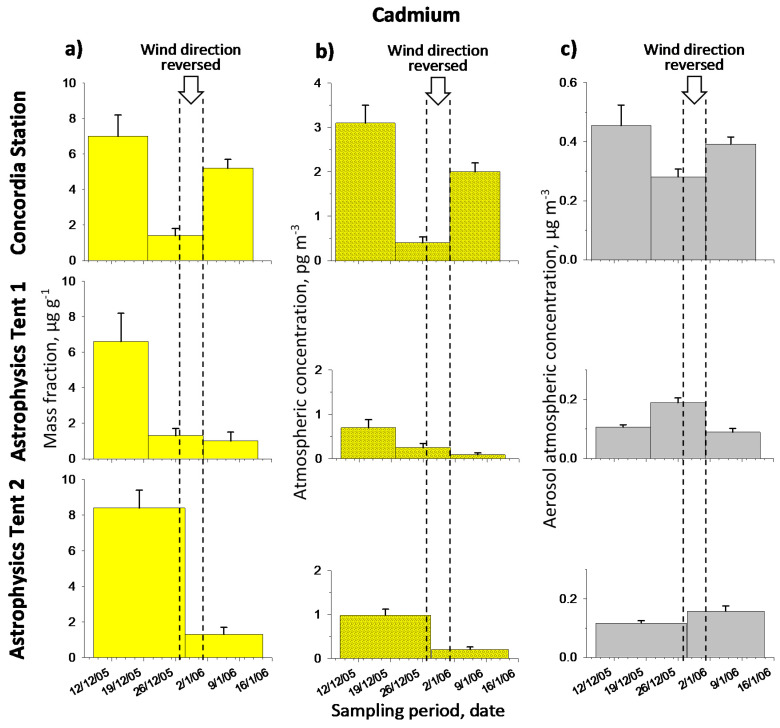
Temporal trends of Cd content at Dome C during the 2005–2006 austral summer, for (**a**) mass fraction and (**b**) atmospheric concentration. In (**c**), aerosol atmospheric concentration reported for comparison (redrawn from [42]).

**Figure 3:**
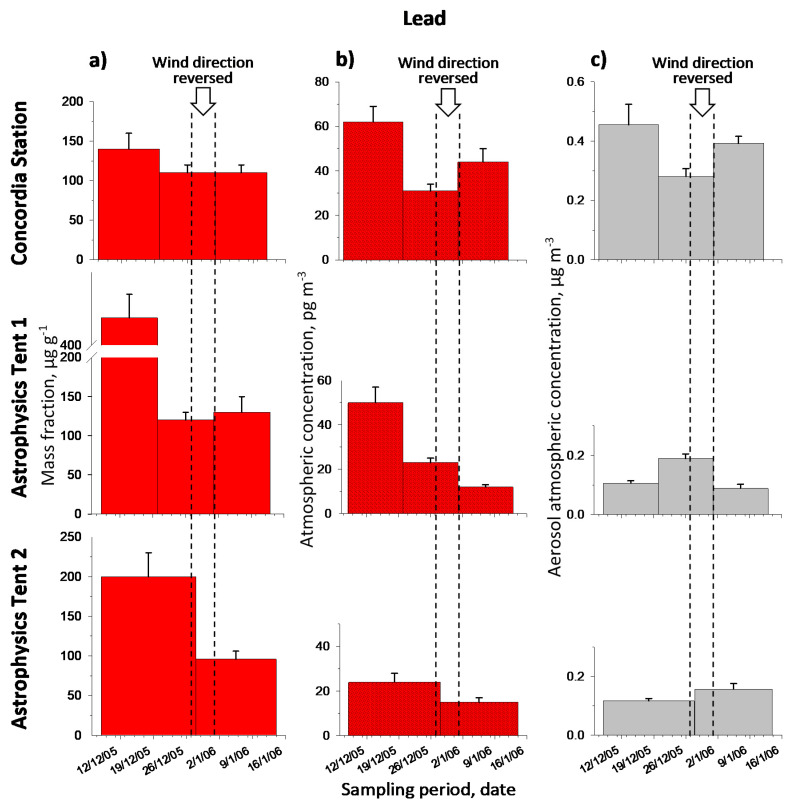
Temporal trends of Pb content at Dome C during the 2005–2006 austral summer, for (**a**) mass fraction and (**b**) atmospheric concentration. In (**c**), aerosol atmospheric concentration reported for comparison (redrawn from [42]).

**Figure 4:**
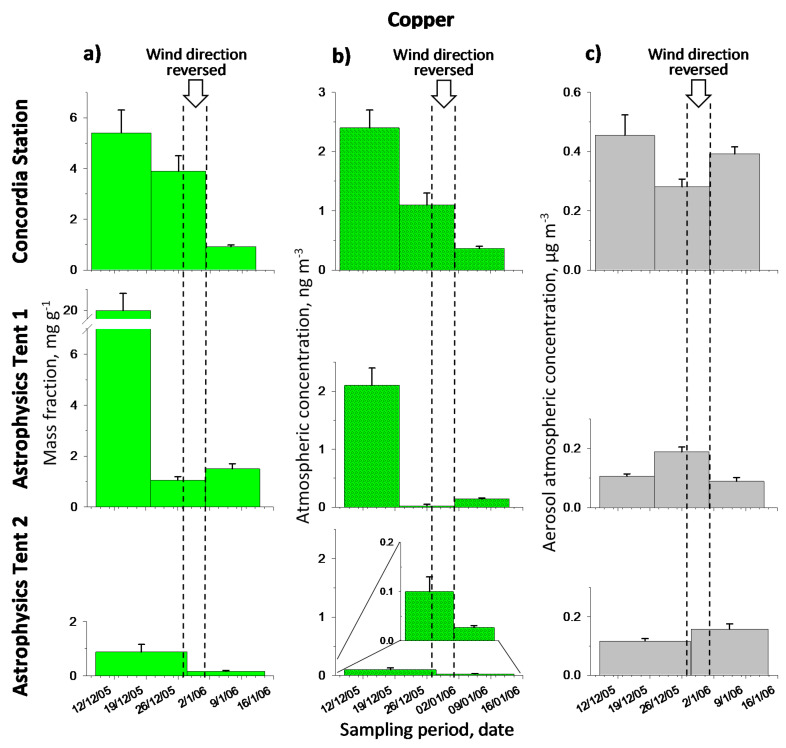
Temporal trends of Cu content at Dome C during the 2005–2006 austral summer, for (**a**) mass fraction and (**b**) atmospheric concentration. In (**c**), aerosol atmospheric concentration reported for comparison (redrawn from [42]).

Table 1 reports the metal mass fractions and the metal atmospheric concentrations, respectively, in the aerosol. Original results are given in Supplementary Materials (Table S9). Note that the metal contents varied, in the period of observations, as follows (min-max). Mass fraction: Cd 1.0–8.4 µg g^−1^, Pb 96–470 µg g^−1^, Cu 0.17–20 mg g^−1^; atmospheric concentration: Cd 0.09–3.1 pg m^−3^, Pb 12–62 pg m^−3^, Cu 0.027–2.4 ng m^−3^. For the latter case, see comparison with literature data in Section 2.3. Figure 2, Figure 3 and Figure 4 show graphically the metal temporal profiles in the two measurement units.

A general analysis of data reported in Table 1 and showed in Figure 2, Figure 3 and Figure 4, suggests that the highest values were observed at the beginning of the expedition (corresponding to sample 1 for each sampling point); this can be attributed to the intense activity at Concordia Station, including aircraft arrivals/departures. A deeper discussion will follow on the single metal profiles, which take account of the information related to the temporal profile of the aerosol atmospheric concentration and to wind direction to evaluate background metal contents and impact of local sources.

#### 2.2.1. Cadmium

Cadmium content (Table 1 and Figure 2) varied in the range 1.0–8.4 µg g^−1^ (0.09–3.1 pg m^−3^) with the highest values observed for the site Concordia, as expected. High values are also observed for the first sample of each temporal profile, independently of the sampling point. These high values can be considered as the consequence of the intense field activity at the station related to the arrival of researchers, together with all the material and its arrangement in the area, needed to set up and to start the research scheduled for the Austral Summer. Much lower values are observed subsequently.

If we consider the complete temporal profiles observed for the two measurement units available, we note for both very similar patterns, for which the same general interpretation can be given. However, on a closer inspection, higher variations are observed when the atmospheric concentration unit is considered rather than that of mass fraction. This behavior is due to the fact that the metal mass fraction (Figure 2a) and the aerosol concentration (Figure 2c), i.e., the two quantities which combine to obtain the metal concentration, vary ‘in phase’ with each other, i.e., they change in the same way (in association or in concordance). Thus, an increase in metal content in the aerosol (mass fraction unit) also corresponds to an increase in aerosol concentration, and the same for a decrease. Therefore, changes in aerosol concentration adds to (combines in concordance with) changes in the metal content (mass fraction) in the aerosol giving as a result more pronounced changes when we pass from data in mass fraction to those in metal atmospheric concentration. For example, if we compare sample no.2 with sample no.3 of the site Concordia, we see that the effect of less aerosol content in sample no.2 with respect to sample no.3 (−28%, see Figure 2c) adds to the effect of less metal in the aerosol (−73%, see Figure 2a) giving a final result of an increased difference in the atmospheric concentration of the metal, which adds up to −80% (Figure 2b), which is higher than the same difference expressed in mass fraction, i.e., −73% (Figure 2a). A similar observation can be made for the site ‘Astrophysics Tent 1’ if we compare sample no.2 with respect to sample no.3. Here, the effect of an increase in metal content (+30%) adds to the effect of an increase also in aerosol concentration (+112%) to obtain a much more difference when we consider the increase in metal atmospheric concentration (+178%) with respect to the increase in mass fraction (+30%). Thus, we can conclude here that a higher aerosol quantity in the air also corresponds to a greater metal content in the aerosol, and that the same association occurs for low quantities; this means that when we have more aerosol, this is also more concentrated in the metal, and vice versa. Then the combined effects of the two factors leads to changes in Cd atmospheric concentration which are amplified with respect to those observed for the mass fraction.

Looking at the profile of site Concordia, we note a clear effect of the wind direction. In fact, very low values, 1.4 µg g^−1^ or 0.40 pg m^−3^, are observed for sample no.2, when, for several days (27 December–1 January) the wind blew from North instead of from South, leaving the sampling point upwind of the Station Concordia, instead of downwind as typical. Then the values turn to increase in the last sample (no.3), 5.2 µg g^−1^ or 2.0 pg m^−3^, when the wind blew in the typical direction from South to North, with emissions from the station impacting directly on the sampling point.

For the data collected at the astrophysics tent, if we exclude the two initial samples, since they suffers of a generalized effect of the beginning of the expedition, we observe here the lowest values ever observed in Central Antarctica, i.e., 1.0–1.3 µg g^−1^ or 0.09–0.25 pg m^−3^. These values can be considered to establish the background level of Cd at Dome C (see below). Even in this case the effect of the reversed wind direction, which happened in a substantial part of the central period of sampling, is clearly visible comparing the sample no.2 with no.3 of Astrophysics Tent 1. In fact higher values are observed for sample no.2, collected when the wind direction blew in part from the station towards the site, than for sample no.3, with wind blowing in the prevailing direction from South, i.e., 1.3 vs 1.0 µg g^−1^ or 0.25 vs. 0.09 pg m^−3^.

In conclusion, we can set the background level for Cd at Dome C by averaging the results obtained for samples no.2 of CS, no.2 and no.3 of AT1 and no.2 of AT2, obtaining the following values: Cd background content in mass fraction 1.2 ± 0.2 µg g^−1^ (range 1.0–1.4 µg g^−1^), and in atmospheric concentration 0.24 ± 0.13 pg m^−3^ (range 0.09–0.40 pg m^−3^). By contrast we can observe for the other, contaminated, samples (CS-1, CS-3, AT1-1, AT2-1) the following statistics: 6.8 ± 1.3 µg g^−1^ (range 5.2–8.4 µg g^−1^), in mass fraction, and 1.7 ± 1.1 pg m^−3^ (range 0.7–3.1 pg m^−3^), in atmospheric concentration. Note here the much higher variability with respect to the background values. We can ascribe this high variability to the more fluctuating nature of local pollution. On average the increase of the metal content on the background (the excess) due to the local pollution is 5.6 ± 1.3 µg g^−1^ (1.5 ± 1.1 pg m^−3^).

#### 2.2.2. Lead

The lead content (Table 1) varies in the range 96–470 µg g^−1^ (12–62 pg m^−3^). The highest values are observed for the first sample of each time series, which, in this case too, expresses an initial temporarily high contamination of the full area, very likely due to the beginning of the summer activities at Dome C. It is to be noted, however, that the absolute highest concentration in terms of mass fraction in this case occurs for the first sample collected in the site of Astrophysics Tent 1 instead of Concordia.

Looking at the temporal profiles of Pb expressed in mass fraction (Figure 3a), we observe here a rather different situation than that of Cd. If we exclude the first sample of each series, all the others show substantially uniform content in the three sites with values in the range 96–130 µg g^−1^, independently of the sampling point and the wind direction. This means that, always excluding the first sample of each series, the Pb content in the aerosol is practically the same independently of the points of sampling, which can represent a generalized background contamination throughout the area, for this element. From this point of view, the aerial photo of the area (see Section 3.1) could well represent the situation of an area substantially affected by local emissions, which partially also includes the sampling points at the astrophysics tent.

With reference to the profiles expressed in atmospheric concentration of Pb (Figure 3b), given the above on the uniform Pb content in the particulate matter, we observe that—always with the exception of the first samples of the series—they reflect only the greater or lesser aerosol content (of substantially the same composition), as observed in Figure 3c, and whose variation is therefore also sensitive to wind direction. Thus, looking at the profiles of CS and AT1 for samples no.2 and no.3, a close relationship can be observed with the wind direction and the connected atmospheric aerosol concentration given in Figure 3c. In fact, for the point CS, when the wind direction reversed for part of the time (sample no.2) with respect to the prevailing one (sample no.3), leaving it upwind of the station, a lower atmospheric concentration of Pb is observed (compare sample no.2 with no.3), which is simply due to a lower aerosol content in sample no.2 with respect to sample no.3 (Figure 3c), since the Pb content in the aerosol (expressed in mass fraction) is approximately the same in the two samples. Obviously, the situation in AT1 is reversed with respect to CS. Here in fact the atmospheric concentration of Pb is higher in the second sample (wind direction reversed and sampling point downwind of the station) than in the third one (wind direction from South and sampling point upwind of the station). Once again, the importance of reporting the data both in mass fraction and in atmospheric concentration emerges even more clearly in this case. In this case, the importance emerges of reporting the data both in mass fraction (which expresses the content of the analyte in the aerosol) and in atmospheric concentration (which expresses the analyte content per unit volume of the air and that is a combination of the content in the aerosol and the quantity of the aerosol which is present in the air).

In conclusion, it does not seem possible in the case of Pb to set up a clear background content for the site Dome C, but rather to fix an upper limit for this background. This upper limit (‘background’) can be estimated, as mass fraction (from samples CS-2, CS-3, AT1-2, AT1-3, AT2-2) in the range of 96–130 µg g^−1^ (or 113 ± 13 µg g^−1^), and as atmospheric concentration (from samples CS-2, AT1-2, AT1-3, AT2-1, AT2-2) in the range of 12–31 pg m^−3^ (or 21 ± 8 pg m^−3^), with minimum values of the order of 12–15 pg m^−3^. By contrast, for the other, contaminated samples, we obtain for mass fraction (CS-1, AT1-1, AT2-1) 270 ± 180 µg g^−1^ (range 140–470 µg g^−1^), and for atmospheric concentration (CS-1, CS-3, AT1-1) 52 ± 9 pg m^−3^ (range 44–62 pg m^−3^). Also note in this case the higher variability of contaminated samples with respect to ‘background’ samples, as already observed and commented for Cd. From these data we can compute an average increase of the metal content on the ‘background’ (the excess) due to the local pollution of 157 ± 180 µg g^−1^ (31 ± 12 pg m^−3^). The very high variability of the first figure is due to an extremely high value of a contaminated sample.

#### 2.2.3. Copper

In comparison to the other two metals, copper shows the highest contents together with greater variability (Table 1). In this case the range is 0.17–20 mg g^−1^ (0.027–2.4 ng m^−3^). Once again, the highest contents occur for the sampling point Concordia, even if, as for Pb, the highest value in absolute can be observed for the first sample of Astrophysics Tent 1.

In general, copper shows a behavior again different from the other metals and also diversified between the three sampling points (Figure 4). In particular considering the CS point, the content remain again high even when, in the second sample, the site itself is upwind of the station for part of the time, and then it decreases in the next sampling when the wind conditions are such that the site is under the direct influence of the station. Somewhat surprisingly, in this case the concentrations of the two sampling points at the astrophysics tent site (AT1 and AT2), despite being only a few tens of meters away from each other, show clearly different values, with sampling point AT2 showing the lowest values.

In this case, the profiles expressed in atmospheric concentration are very similar to those in mass fraction and in any case strongly conditioned by the latter, with no simple and easily interpretable effect of the wind direction. This suggests that some other extemporaneous and not clearly identified factor may have intervened in this case to complicate the trends.

Concerning the copper background content of the study area in terms of mass fraction, if we exclude the first two samples of CS and the first one of AT1, we have a range of 0.17–1.5 mg g^−1^ or 0.91 ± 0.48 mg g^−1^. Referring instead to the atmospheric concentration of Cu, it seems more reasonable to exclude also sample no.3 of CS obtaining a range of 0.027–0.20 ng m^−3^ or 0.12 ± 0.07 ng m^−3^. Conversely, for the other, contaminated samples, we obtain for mass fraction (CS-1, CS-2, AT1-1) 9.8 ± 8.9 mg g^−1^ (range 3.9–20 mg g^−1^), and for atmospheric concentration (CS-1, CS2, CS-3, AT1-1) 1.49 ± 0.94 ng m^−3^ (range 0.36–2.1 ng m^−3^). We note here again, as before, the much higher variability of contaminated samples with respect to background samples. From these data we can compute an average increase of the metal content on the background (the excess), i.e., the amount possibly due to the local pollution, of 8.9 ± 8.9 mg g^−1^ (1.37 ± 0.94 pg m^−3^). Also, in this case the very high variability of the first figure is due to an extremely high value of a contaminated sample.

#### 2.2.4. Relationship between Contaminated-on-Background Excesses of the Three Metals

A useful indicator for the metal sources can be obtained from the previous results on metal excesses of contaminated on background samples. Thus, the mass ratio between excesses of the three metals can be computed approximately as follows (with large variability):

- excess mass ratio from content in mass fraction Cd:Pb:Cu = 1:28:1600 (with Cu/Pb = ~57);
- excess mass ratio from content in atmospheric concentration Cd:Pb:Cu = 1:21:800 (with Cu/Pb = ~38).

### 2.3. Comparison with Antarctic Literature Data

A comparison of present Dome C results with other Antarctic data can be found in Table 2. Here available literature data are reported with reference to both the Antarctic Continent (inland area and coastal sites) and the Southern Ocean. For the present study, the reported averages are computed excluding samples whose high values were attributed to local pollution, as discussed above. It can be seen from the table that, as already mentioned, no other data are available for the Dome C site, and that even for the plateau areas of Central Antarctica only the South Pole site is present. Moreover, in no cases are results expressed in mass fraction available for Antarctic sites.

In general, if we exclude our present results of Dome C, it can be observed that, given the great variability of the data and the fact that those referring to the central area of the continent concerns only South Pole, and with generally old measurements, no differentiations can be observed between metal contents detected in the plateau area and those reported for coastal sites, and even for the Southern Ocean. Anyway, certainly metal atmospheric concentrations increase in the order Cd < Pb < Cu passing from a few pg m^−3^ to several ng m^−3^.

For cadmium, it can be seen that our results are the lowest ever reported for inland sites highlighting that background Cd concentration in plateau regions may be well below the previously reported values, and that is around a few tenths of pg m^−3^ instead of a few units or tens of pg m^−3^.

As regards lead, our measurements indicate upper limits for background values of some tens of pg m^−3^, which are located at the lower end of the other data referred to both inland and coastal site, even if literature data show frequently much higher values, up to several hundreds of pg m^−3^ (see e.g., results reported for McMurdo, the Southern Ocean, and various others).

Our copper data stay for a background value of around one hundred pg m^−3^ (with the lowest result fixed at 27 pg m^−3^) which is in agreement with data reported for South Pole by Cunningham and Zoller [34], and by Tuncel et al. [35], but a little higher of that reported by Zoller et al. [31], Maenhaut and Zoller [32], and Maenhaut et al. [33], for the same site, which stay for a few tens of pg m^−3^. Generally, much higher concentrations are observed for coastal sites and for the Southern Ocean, where values of several hundred pg m^−3^ are typical.

### 2.4. Statistical Analysis (Correlation and Principal Component Analysis)

To deepen and better understand the relationships between metals and between metals and samples, we carried out a (bivariate) correlation/regression analysis (CA, RA) and a (multivariate) principal component analysis (PCA, see e.g., [134]).

**Figure 5:**
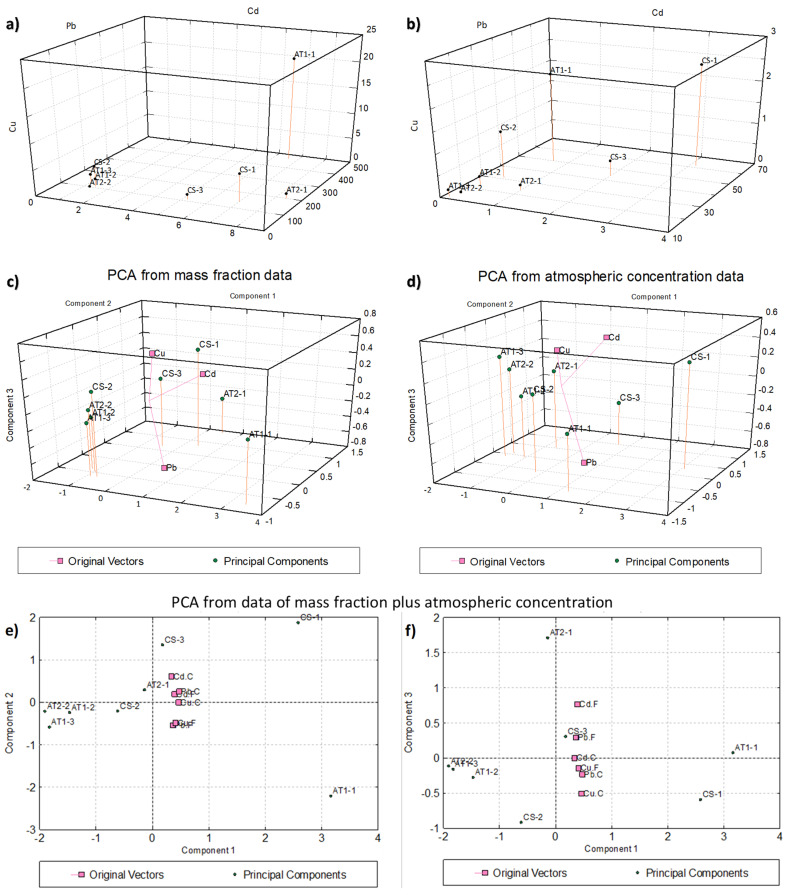
(**a**,**b**) 3D plots of original data in (**a**) mass fraction (Cd and Pb in µg g^−1^, Cu in mg g^−1^) and (**b**) atmospheric concentration (Cd and Pb in pg m^−3^, Cu in ng m^−3^). (**c**,**d**) 3D PCA biplots from data in (**c**) mass fraction and (**d**) atmospheric concentration. (**e**,**f**) 2D PCA biplots from data in mass fraction (Cd.F, Pb.F, Cu.F) and atmospheric concentration (Cd.C, Pb.C, Cu.C) on the planes of (**e**) PC1 vs PC2 and (**f**) PC1 vs PC3. Squares: original vectors (PC loadings); circles: PC scores.

Concerning CA and RA, in a first step we considered all samples, independently of the sampling point and time, to have a general scenario of metal associations from the whole data set. Then, considering that the previous discussion (Section 2.2) showed that there are two well-defined data groups (which approximately represented background and contaminated samples, respectively), separated analyses were carried out for each of these two groups individually. Given the bivariate nature of correlation we had to include in each group the same set of samples for all the three metals, independently of differences showed above from one metal to another, especially to define background values, and also, for the same metal, between measurement units. To make the most appropriate selection, we observed the 3D plots of original data, the 3D PCA biplots obtained from mass fractions and from atmospheric concentrations, and the 2D PCA biplot obtained from both measurement units (details on PCA procedure and results in Supplementary Materials, Section S2, Tables S1–S6, Figures S2–S4). The resulted plots, reported in Figure 5, suggested us to make the following choice for the two groups to be used for separate background/contamination correlations: background samples CS-2, AT1-2, AT1-3, AT2-2 (even if sample CS-2 represents a borderline situation were the Concordia sampling point was upwind of the station only for part of the sampling time); contamination samples CS-1, CS-3, AT1-1, AT2-1.

**Figure 6:**
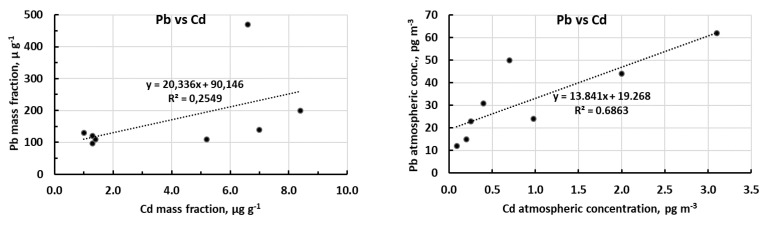

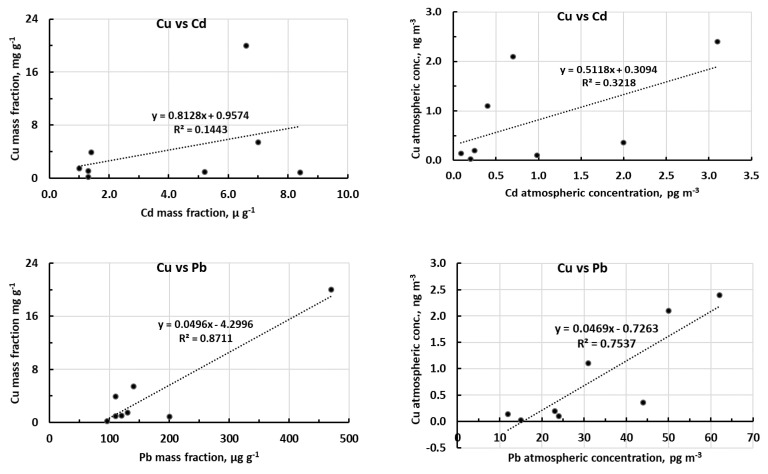
Bivariate scatter plots of metal contents (in mass fractions and atmospheric concentrations) in the aerosol samples collected at Dome C during austral summer 2005–2006.

The Table 3 reports the correlation coefficients calculated both for mass fraction and atmospheric concentration, while Figure 6 shows the related scatter plots obtained from the corresponding regression analyses.

The most significant correlation (*p*-value < 0.05) is between lead and copper, both in mass fraction and in the atmospheric concentration. Cadmium and lead show a high correlation (at the limits of significance) only for the atmospheric concentration (*p* = 0.06).

As for PCA (details in Supplementary Material, Section S2), we mention briefly here that results of the three runs all show that the first PC collects the most part of the total variation (62–84%) and expresses the overall metal loads, while the second component broadly separates and contrasts Pb and Cu on one side and Cd on the other side, even if PC2 is significant only for the elaboration with both measurement units as variables.

The results of separate correlation/regression analyses carried out for the two subsets identified above are reported in Table 4 and, in graphical form, in Figure S5. Again, the most relevant correlation regards the Cu vs. Pb relationship especially for the subset of contaminated samples. In this analysis, to give further evidence of the possible differentiation between local (contamination) and remote (background) metal sources, the regression coefficients (slopes of the regression lines) of the two subsets were compared with those of the full data set, and the changes used as possible discriminating signals (markers). From this comparison (see Table 5 in numerical form and Figure S5 at glance) it can be seen that the regression coefficients for the ‘contamination’ series broadly follow (with only one exception) those of the full dataset. Conversely, regression coefficients for the ‘background’ series show (again with only one exception) very different slopes both each other and with respect to the full dataset; indeed, they do not show any regularity, and they change almost casually.

Despite that the eight available samples (and much more the four samples for the two subsets) are rather low numbers, the data analyses reported above reasonably suggests that some of the samples are representative of a scenario where the station activity is the main factor responsible for metal concentration in the aerosol, while other samples are representative of a scenario classifiable as ‘background’, where metal concentrations in the aerosol are representative of the general air masses circulation.

### 2.5. Metal Contents in Station Fuels and Interpretation of Data

To support the hypothesis of the main contribution of local emissions for the high-value data series, we analyzed all the three main fuels used in the site, i.e., the SAB diesel fuel, the unleaded gasoline, and the Jet A-1 fuel. The results of metal contents in the three fuels are reported in Table 6, together with the ratios between metals. It can be seen that data reported are in the general range of values found in the literature [135,136,137,138,139,140,141,142,143,144,145]. However, for data interpretation we used only results obtained for SAB, which covers the vast majority of the fuel consumed at Concordia Station, since the other two fuels are used in very much smaller quantities. From these data a metal mass ratio Cd:Pb:Cu of 1:14:100 can be observed for the SAB diesel fuel. This ratio, with the exception of the value involving Cu, is broadly comparable with those obtained for metal excesses of contaminated on background samples—i.e., Cd:Pb = 1:21 or 1:28—from atmospheric concentration or from mass fraction, respectively (see Section 2.2.4), and from regression coefficients of metal pairs from contaminated samples (9–20 µg_Pb_/µg_Cd_ for Pb vs. Cd in Table 5). Note that the very high values of Cu mass ratios in contaminated samples (e.g., 800–1600 for Cu:Cd, Section 2.2.4), or regression coefficients (about 400–800 µg_Cu_/µg_Cd_ for Cu vs. Cd Table 5) cannot be explained by the only effect of emissions from SAB fuel consumption. Since some backward trajectories come from the West Antarctica, particularly the Antarctic Peninsula (Figure S1), we can argue that this fact could be the results of particularly intense atmospheric events in that area or the southern South America; however we cannot prove this specific hypothesis, given the low temporal resolution of our data. Note again that, as expected, the metal ratios for the other two fuels are effectively very different from the indicator metal ratios obtained from the ‘contaminated’ samples (metal excesses and regression coefficients).

Therefore, considering first the series of aerosol samples which show high contents of all the metals, we can reasonably conclude that this enrichment with respect to the other (background) series is the result of the local pollution from the logistics of the expedition, mainly due to the emissions of the two electrical power generators of the site (Concordia Station and Dome C ‘Summer Camp’), but also by the various means of transports used on site, such as snowcats, snowmobile, aircrafts, and the vehicles of the ITASE transport traverse from Dumont d’Urville to Dome C.

If we then look at the second series of aerosol samples—i.e., the one related to the hypothesized background values—we observe that no metal ratio obtained for the SAB diesel fuel can be associated to the same ratio computed for metals in the aerosol, as those obtained from regression coefficients (compare data on Table 6 with those on Table 5). This result allows us to conclude that the metal contents of this series of samples cannot be related to local pollution and, consequently, that such data can be effectively associated to background values in the area of Dome C due to transport of atmospheric particulate matter from remote sites to the Continent, or from other sites of Antarctica to Dome C, and then representative of the general air mass circulation over the Antarctic plateau.

Referring to Cd, in the recent literature its presence in the Antarctic environment is usually associated with anthropic origin [47,146,147], mainly since in the crust it is present at very low concentrations [46] and practically absent in Antarctic ores [92,93]. However, a volcanic origin of the metal should also be considered. Indeed, on a global level it has been estimated that volcanic emissions contribute about 40% of the Cd annually emitted into the atmosphere [61], while for Antarctica several studies reported on this source for Cd [30,60,115,121,148].

Similarly, also for Pb the main cause of its enrichment in Antarctica is associated to remote sources of industrial emissions, especially from mining and smelting activities [74], and, in the recent past, from emissions due to the use of leaded gasoline as engine fuel [76,149], or in any case with anthropogenic pollution [2,45,47]. In fact, in the Antarctic crust it is present at low concentration, mainly in association with copper minerals and with silicic volcanic materials [92,93].

As for Cu, although often the presence of the metal in the Antarctic atmospheric aerosol is attributed mainly to anthropic origin [2,14,45,47]—here, in the East Antarctic Plateau—it may also have an important natural crustal origin. Indeed, as reported in the introductory section, along the east coast of the Antarctic Peninsula there are many copper ores, also in rocks not covered by ice, and dusts coming from Patagonia and Tierra del Fuego, rich in copper, have been demonstrated to have an influence on the metal enrichment of Antarctic aerosol trapped in ice cores (see detailed discussion and references in Section 1). Moreover, as shown above by 5-day back trajectories, the air masses arriving at Dome C passed through the western coastal area in front of the Antarctic Peninsula, where there are copper minerals in the rocks. Such scenario, together with the study on wind back trajectories, supports the hypothesis that copper in background samples may have remote anthropic and natural origins.

## 3. Materials and Methods

### 3.1. The Site and the Sampling Strategy

Dome C (Concordia Station), 75°06′ S, 123° 21′ E, is a remote site on the East Antarctic continental plateau, located at an altitude of 3233 m a.s.l. and more than 1000 km from the coastline. This place has been defined among other things as “The Best Site on Earth for the 21st Century Astronomers” [150]. Here the climatic conditions are really extreme, and the average values are as follows: air temperature in summer −30 °C (−60 °C in winter with a minimum of −84.6 °C), wind speed 2.8 m s^−1^ (5.4 knots) with a maximum of 17 m s^−1^ (33 knots), atmospheric pressure 645 hPa, annual precipitation 2–10 cm of snow, prevailing wind direction from the South in summer, relative humidity 55%.

**Figure 7:**
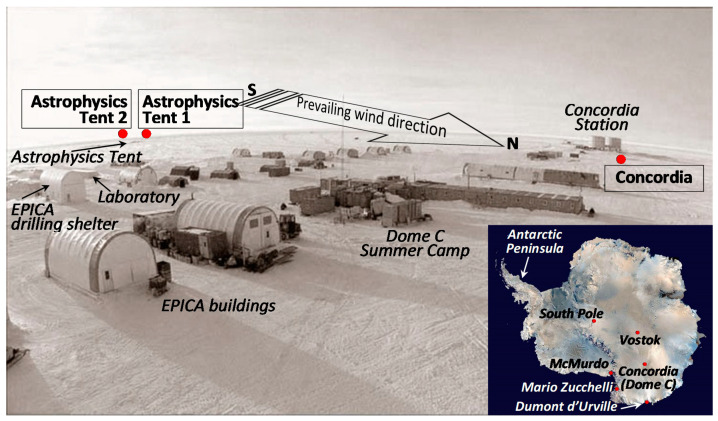
View of the Dome C area through a wide-angle panoramic photo (with Concordia Station in the background and Dome C-Summer Camp in the foreground). The insertion shows the location of Dome C on a map of Antarctica. The photo shows also the sampling points, big red circles (Concordia, CS; Astrophysics Tent 1, AT1; Astrophysics Tent 2, AT2) and the prevailing wind direction (from south to north) referred to the sampling period.

**Figure 8:**
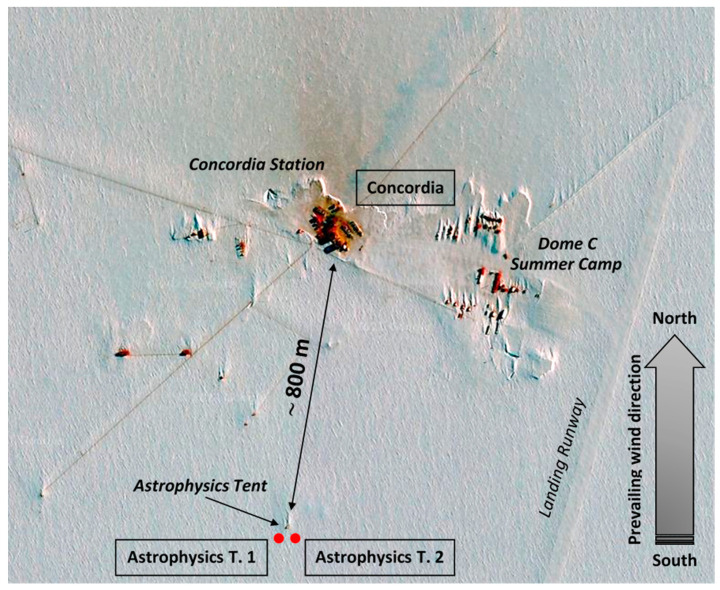
Satellite image of the Dome C area showing, among other things, the sampling points, the typical drift patterns of windblown snow around buildings and the pollution influence of the Concordia Station (from Google Earth).

During the 2005–2006 Italian austral summer expedition in Antarctica, atmospheric aerosol was collected by one of the authors (GS) at Dome C (Figure 7 and Figure 8, Figure S6) from 7 December 2005 to 14 January 2006, with the aim to determine the total contents of Cd, Pb, and Cu [42]. Sampling was carried out using three PM10 high-volume impactors installed in two sites. One sampling unit was located immediately downwind of Concordia Station (CS), ~50 m to the north, and it is referred to as Concordia, or simply CS (Figure S7a). The other two systems were located close each other (~50 m apart), near the astrophysics tent (cited as AT1 and AT2), at about 800 m south (upwind) of the Station and ~150 m south of the tent (Figure S7b). The astrophysics tent was not used during the period of sampling; thus, this ‘distant’ site was considered to be a ‘clean’ site. The exposure time was set to comply with the requirements of trace element determinations, and differentiated between the three samplers, given the total absence of previous information on the metal contents. At Concordia Station and at Astrophysics Tent 1, each sample was collected for about ten days, while at Astrophysics Tent 2 the sampling period was extended to about 20 days. This strategy was adopted since at that time we were not able to estimate the actual metal contents, thus if a 10-day exposure had not be enough to detect ultra-trace metals, at least the second sampler on the astrophysics tent site could have been adequate to collect enough material to have metal contents above the detection limits. The periods of exposure (December 2005 through January 2006) were as follows: (i) CS and AT1, 7 (8 for AT1) December–20 December, 20 December–1 January, 1 January–12 January; (ii) AT2, 8 December–28 December, 28 December–14 January. The exact air volumes passed through the filters during each exposure, the aerosol masses measured, and the corresponding aerosol atmospheric concentrations computed (expressed in µg m^−3^), are reported elsewhere [42].

Blank filters (‘field blanks’) were also collected in the field for each sampler, both at the beginning of the activity and at the end of the sampling program. They were not exposed to air flux, but simply installed on the switched-off samplers for a few tens of minutes and then treated like sample filters.

The location of Dome C in Antarctica together with sampling points in the area is shown in Figure 7, while the technical map in Figure S6 gives a more precise and detailed view of the sampling site. The prevailing wind direction during the sampling activity, i.e., from south to north, is also depicted in the two figures. A satellite image of the Dome C area with sampling locations highlighted is also reported in Figure 8 (note that here the south–north direction is reversed with respect to previous two). The typical drift patterns of snow around isolated buildings highlight snow deposits accumulated downwind of buildings (northward), which confirm the south–north prevalent wind direction. Moreover, a brown area (a sort of cloud or plume) is also visible around the station, but mainly directed from the station northward. This plume points out the atmospheric pollution due to the influence of Concordia Station and of the other buildings. No clear pollution evidence is visible in the ‘clean site’ of the astrophysics tent.

During the period of sampling the science station hosted 20–40 people and about 35 kL of special Antarctic blend (SAB) diesel fuel were used during the 39 days of sampling. A very much lower, insignificant, consumption regarded the use of unleaded gasoline fuel (only for skidoos, not quantified) while the Jet A-1 fuel (on average ~38 kL for a full summer campaign) was in fact used exclusively to refuel the Twin Otter airplane and therefore it is virtually all burned elsewhere and only to a very minimal part consumed on site. About 10 Twin Otter flights arrived at Dome C during the fieldwork. One logistic traverse of the Italo-French International Trans-Antarctic Scientific Expedition (ITASE) from Dumont d’Urville to Dome C arrived at Dome C and remained a few days between the end of December and the beginning of January.

### 3.2. Meteorology and Air Backward Trajectories

Data and information on local meteorology were obtained from the Antarctic Meteo-Climatological Observatory (http://www.climantartide.it/ accessed on 31 March 2021) funded by the Italian National Programme of Antarctic Research (PNRA, Programma Nazionale di Ricerche in Antartide) and managed by staff of the Italian National Agency for New Technologies, Energy and Sustainable Economic Development, section Antarctica Technical Unit (ENEA-UTA) (http://www.uta.enea.it/ accessed on 31 March 2021).

**Figure 9:**
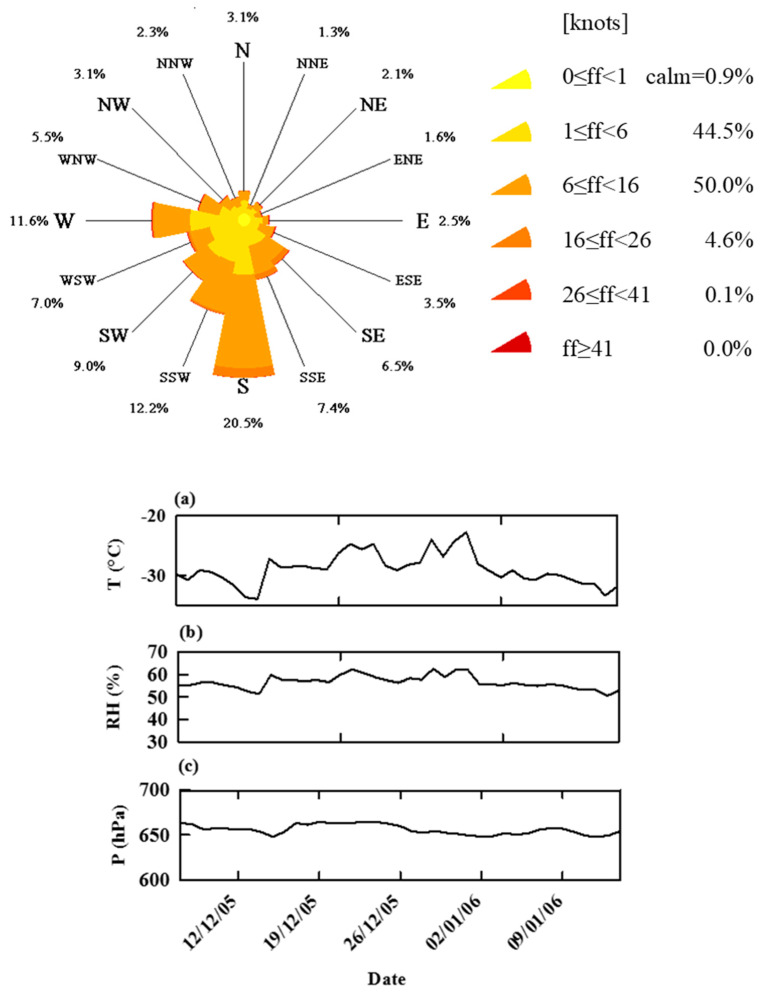
Wind rose and meteorological data at Dome C during the sampling period. (**a**) Air temperature T (°C), (**b**) relative humidity RH (%), (**c**) pressure P (hPa).

Meteorological parameters (air temperature, relative humidity, ambient pressure, wind speed, and direction) were continuously registered at time intervals of 1 h by the Automatic Weather Station (AWS) located close to the sampling sites at Concordia Station. The mean values of these parameters are reported in Figure 9 together with the wind rose. During the sampling period the prevailing wind direction was, as expected, from south to north, except from 27 December to 1 January when it was reversed. Average meteorological data were as follows: temperature −29 °C, pressure 656 hPa, and relative humidity 57%.

In order to characterize the transport pathways of air masses arriving at Dome C, backward air trajectories were computed using the Hybrid Single-Particle Lagrangian Integrated Trajectory (HYSPLIT, version 4) transport and dispersion model accessed and interactively ran via the National Oceanic and Atmospheric Administration (NOAA) Air Resources Laboratory’s (ARL) READY (Real-time Environmental Applications and Display sYstem) web site http://www.arl.noaa.gov/ready/hysplit4.html accessed on 31 March 2021, [151,152]. The meteorological data used for the calculations were from the NOAA Global Data Assimilation System (GDAS) of the National Center for Environmental Prediction (NCEP), 1° by 1° grid. The vertical motion calculation method was selected to model vertical velocity. The back trajectories were calculated for each day (arrival time 12 am), going back 5 days earlier with a 6-h interval time. Three arrival heights were selected, 100 m, 500 m, and 1000 m above ground level (a.g.l.), to minimize ground friction and mixing air processes close to the soil.

The choice of 5 days was made due to the mean lifetime expected for aerosols in this region. In fact, the residence time of aerosol is usually less than a week, depending on aerosol properties and meteorological conditions [13,43,153,154,155].

### 3.3. Laboratories, Apparatus, Reagents, and General Procedures

#### 3.3.1. Laboratories and Apparatus

Clean room laboratories, with areas under laminar flow cabinets in Class 5 (ISO 14644-1; formerly Class 100, US Fed. Std. 209e), were available both in Antarctica, at Mario Zucchelli Station [29] and at Concordia Station [42], and in Italy [30,156,157]. All the most critical procedures connected with decontamination of materials, sample treatment, and sample analysis, were carried out in these laboratories. At Concordia Station the laboratory temperature was set to 15.0 ± 0.5 °C, while relative humidity (RH) naturally resulted 2.5% ± 0.5%, without any further control. To obtain these conditions the scientist was inside the climatic room only for the strict time necessary for the processing of the samples; in particular the weighing procedure was managed by computer from the outside the clean room with the computerized microbalance inside. In Italy the temperature is set at 20 ± 1 °C during the winter and 23 ± 1 °C during the summer, with RH 50% ± 1%. Inside these laboratories, scientists, and other personnel strictly followed clean room procedures, always wearing clean-room garments, masks, and gloves.

Three Teflon-coated (inner and outer), high-volume (1.13 m^3^ min^−1^ ± 10%), impactor-based, brushless samplers (model TE-6070V-BL, from Tisch, Village of Cleves, OH, USA) were used for collection of PM10 atmospheric aerosol samples. Before use, in Antarctica, the samplers (impactors) were cleaned inside and outside (Section 3.3.3). Afterwards they were calibrated in the field, outside of the Antarctic laboratory, both at the beginning of the activity to assure accuracy before sampling and at the end of the experiments for the final a posteriori verification [42].

Filters for PM10 collection were acid-cleaned 8 × 10 in (20.3 × 25.4 cm) ashless Whatman 41 cellulose filters (cat. no. 1441-866, thickness 220 µm, ashes ≤ 0.007%, pore size 20–25 µm, basis weight 85 g m^−2^), which were specifically prepared and tested for trace element determinations (Section 3.3.3).

Computerized microbalances from Mettler Toledo (Greifensee, Switzerland) were used in Antarctica (model AT261) and in Italy (model XS205), both with readability 0.01 mg and repeatability SD = 0.015 mg. Accuracy tests for the balances were obtained by two certified reference weights (OIML class E1) of 10 mg (certified mass 0.0100005 g, 2SD = 0.0020 mg) and 100 mg (certified mass 0.0999979 g, 2SD = 0.0020 mg).

A microwave (MW) accelerated reaction system MARS 5 (magnetron frequency 2450 MHz) from CEM (Matthews, NC, USA) was used for complete digestion of aerosol filter samples to obtain total metal contents after voltammetric analysis. MW vessels were 100-mL HP-500 plus in Teflon PFA (perfluoroalkoxy copolymer) from CEM.

The voltammetric instrumentation used for (total) metal determinations consisted of a Metrohm (Herisau, Switzerland) 746 VA Trace Analyzer and a 747 VA Stand, equipped with a Teflon PFA (perfluoroalkoxy copolymer) cell and a three-electrode system, which includes an epoxy-impregnated graphite (Ultra Trace graphite) rotating disk working electrode (to be used, after Hg deposition, as a rotating thin mercury film electrode, TMFE), an Ag/AgCl, KCl 3 mol L^−1^ reference electrode (to which all potentials are referred throughout) and a glassy carbon rod counter electrode. It is used for Cd, Pb, and Cu determinations.

Plastic containers of low-density polyethylene (LDPE) were from Kartell (Milano, Italy). They were used for the storage and transport of filters, as well as for storage of digested and undigested sample solutions. Variable-volume micropipettes and neutral tips were from Brand (Wertheim, Germany), model Transferpette.

#### 3.3.2. Reagents and Standards

The laboratory detergent solution was RSB-35 from Backer (Phillipsburg, NJ, USA) 1 + 100 diluted with pure laboratory water. Ultrapure water was Milli-Q from Millipore (Bedford, MA, USA). Analytical grade HNO_3_ (65%) and HCl (37%) were from Carlo Erba (Milan, Italy). Superpure HCl was of Suprapur^®^ grade from Merck (Darmstadt, Germany). Ultrapure acids (HNO_3_ 70%, HCl 34.5%, HF 47–51%) and ultrapure H_2_O_2_ (30–32%) were of UpA grade from Romil (Cambridge, UK). Superpure Al_2_O_3_ was from BDH (Poole, England), ultrapure KCl was of trace select grade from Sigma-Aldrich (St. Louis, MO, USA), superpure hexadistilled Hg was of Suprapur^®^ grade (purity ≥99.999%) from Merck. Standard solutions of Cd, Pb, and Cu were prepared every two weeks by direct dilution of AAS standards 1000 ± 2 mg L^−1^ (from Carlo Erba) using acidified ultrapure water (2 mL ultrapure HCl brought to 1000 mL final solution with ultrapure water). Research-grade nitrogen (purity ≥99.999%) was from Sol (Monza, Italy). The Certified Reference Materials were: (i) NIST 1648 and 1648a urban particulate reference materials, from National Institute of Standards and Technology (NIST, Gaithersburg, USA) [158,159]; (ii) NASS-6 open ocean seawater, from National Research Council of Canada (NRCC, ON, Canada) [160].

#### 3.3.3. Decontamination Procedures

Description of the extensive decontamination procedures generally used in our laboratory for LDPE bottles and all other general plastic material are reported elsewhere [30,156]. Briefly, a careful and prolonged washing procedure was applied to new containers with repeated treatments using, in sequence: tap water, diluted detergent solution to remove possible coarse residual, pure laboratory water, and ultrapure water (each washing repeated three times), 1 + 10 diluted analytical grade HNO_3_ in immersion for two weeks, 1 + 10 diluted superpure HCl in immersion for a week (this step repeated two times) and finally 1 + 1000 diluted ultrapure HCl leaved inside for storage, until use. Before use, three washes and rinses were carried out with ultrapure water. A similar procedure was used for PFA microwave vessels, except that the final washing was carried out with a solution of HNO_3_/HF (5 mL ultrapure HNO_3_, 1 mL ultrapure HF and dilution to 100 mL with ultrapure water). These, however, were then repeatedly treated with digestion solution for 1-h cycles and each time subjected to MW digestion. For both kinds of containers, the cleaning procedure continued until values comparable to voltammetric instrumental blanks were reached.

As for impactors, before use, in Antarctica, they were cleaned inside and outside by washing with the detergent solution and then by repeated rinses using laboratory water, while ultrapure water was used for the most critical part in contact with filters and the air flux.

Cellulose filters for aerosol collection were specifically prepared for trace element determinations. The filters were carefully decontaminated with repeated cleaning cycles as follows: washed with ultrapure water (24 h) and decontaminated with 1 + 1000 diluted ultrapure HCl (24 h). At each stage una sample of the washing solution were analyzed to check the progress of decontamination. The procedure is repeated until undetected concentrations are obtained. At the end of the decontamination cycles, the filters were rinsed with ultrapure water, dried under ISO Class 5 laminar flow cabinets (48–72 h) and stored in acid-cleaned plastic bags until use in Antarctica. The results of an experiment, reported in Table S7, show that typically three decontamination cycles were sufficient to obtain no metal signals.

### 3.4. Sample Treatments

In Antarctica, after collection, sample and blank filters were conditioned for a few days under the Class 5 vertical cabinet and weighed in the climatic room equipped with continuous temperature/humidity monitoring and control (Figure S8a). A differential weighing procedure was used to determine gravimetrically the aerosol mass directly from the difference between the mass of the exposed filters and their mass measured before exposure. Details of the procedure and the resulted aerosol atmospheric concentrations obtained for each sampling stage have already been reported [42]. After gravimetric measurements, the filters were subdivided between participating laboratories. For the sampling points Concordia and Astrophysics Tent 1, the whole filters were reserved for our laboratory; while for Astrophysics Tent 2, since the activity was carried out in collaboration with other two research groups, we obtained an aliquot of one-third of each filter collected. After weighing each filter reserved for our work was put into 500 mL decontaminated LDPE bottles, stored at −20 °C, and transported frozen to Italy for subsequent analytical determinations of trace metals Cd, Pb, and Cu.

In Italy, an aliquot (1/8 or 1/12) of the original filters was cut in the clean room laboratory under laminar flux benches (Figure S8b) and then subjected to a microwave-assisted digestion, as specifically set up previously for the subsequent voltammetric analysis [53]. Mineralization was carried out mixing the filter aliquot inside a microwave vessel with 5 mL ultrapure HNO_3_, 1 mL ultrapure H_2_O_2_ and 1 mL ultrapure HF to ensure total dissolution of the filter and complete oxidation of the organic matter, which interferes in the voltammetric measurements. Digestions were carried out using the “ramp to temperature” mode, with pressure limit constraints, in two steps. Vessels were left cool naturally to room temperature, then they were vented, opened and the solutions were diluted to 100 ml using ultrapure water and, in this form, subjected to voltammetric analysis.

Fuel samples (special Antarctic blend diesel fuel, unleaded gasoline, and Jet A-1 fuel) were digested according to the microwave procedure recommended by CEM on the Application Notes for Acid Digestions; Sample Category: Oils and Plastics; Sample Type: Diesel Fuel; Sample Type: Kerosene (same procedure for both fuels). Here a 120-µL sample was poured into a MW vessel, a 10-mL ultrapure HNO_3_ aliquot was added and the sample allowed to predigest by standing open for about 30 min before sealing vessels and proceeding to the heating program (two vessels at a time maximum) as follows: mode “ramp to temperature”; power level 400 W with 100% maximum; ramp time 15 min; pressure limit 800 psig; temperature 200 °C; hold time 15 min. At the end the vessels were allowed to cool freely to room temperature, then they were diluted to 100 mL in decontaminated containers and stored until voltammetric analysis, which was carried out with the same procedure descripted for aerosol measurements. A 10-mL aliquot of ultrapure HNO_3_, which was treated like the samples, was used for the determination of the blanks.

### 3.5. Voltammetric Analysis

The voltammetric determinations of Cd, Pb, and Cu were carried out in the diluted digested solutions inside the clean chemistry laboratory in Italy (Figure S8b).

Detailed descriptions of the electrochemical pre-treatment of the graphite electrode, the thin mercury film electrode preparation and the overall procedure used for SWASV measurements in background subtraction mode has been reported previously [30,53,157,161,162].

For the voltammetric measurements, the three metals were determined separately employing different deposition potentials, while the deposition times varied, due to the metals involved and the concentrations detected, from 10 s to 45 min. The applied voltammetric experimental conditions, which were optimized previously [53] and here modified for the present conditions (Dome C vs. Terra Nova Bay samples), were as follows (Figure S9). The volume of outgassed digested sample solution was always 10 mL. The instrumental parameters for the accumulation step, i.e., deposition potential (*E*_d_) and deposition time (*t*_d_), were: Cd *E*_d_ = −1100 mV, *t*_d_ = 3–20 min (up to 45 min when 1/12 of filter was used); Pb *E*_d_ = −900 mV, *t*_d_ = 3–5 min (up to 15 min when 1/12 of filter was used); Cu *E*_d_ = −900 mV, *t*_d_ = 10–60 s (up to 10 min when the sample with the lowest Cu content was analyzed and 1/12 of filter was used). The subsequent anodic scan always started from the deposition potential ending to +70/+80 mV in each case. The SWASV conditions for this stripping step were *E*_sw_ (SW amplitude) 20 mV, *f* (SW frequency) 150 Hz, step height (Δ*E*_step_) 8 mV, waiting time (*t*_wait_) 60 ms, number of SW cycles superimposed on each potential step 4 (one for conditioning, preparation cycle, and three for analysis, measurement cycles, with the average value used as analytical signal), step time (*t*_step_) 100 ms, delay time (*t*_delay_) 1.5 ms, current sampling time (*t*_meas_) 1.8 ms. Note that the instrument samples and integrates the current over the period *t*_meas_ of the pulse, and that the obtained electrical charge is divided by *t*_meas_ to obtain the average current over the sampling time; this current corresponds to the measurement at the time point *t*_m_ in ms, which is related to *t*_delay_ and *t*_pulse_ as reported in Figure S9. The background voltammograms (to be subtracted from the analytical voltammograms), were registered immediately before sample analysis as follows. An equilibration potential (*E*_equil_) equal to the deposition potential was applied with electrode rotation on, for an equilibration time (*t*_equil_) of 0.5 s for Pb and Cu and 1.0 s for Cd, to which a SW anodic scan followed using the same parameters as in the specific sample analysis. Typical voltammograms can be found elsewhere [30,53].

Quantification was achieved using the multiple standard addition method, and atmospheric concentrations refer to standard air (298 K, 760 mmHg). Original data are reported in Table S9.

### 3.6. Quality Control and Statistical Analysis

The laboratory blank for the three metals, including instrumental blank, blanks from containers and the various solutions used for treatments, blanks from digestion procedures, and filter blanks, have already been reported previously [53]. It was concluded that the overall laboratory blank, even if detectable, is very low with respect to the blank due to filters. These in fact, despite their residual metal contents after cleaning are at ultra-trace levels, contribute significantly because of their very much greater mass of the cellulose material with respect to the aerosol mass (typically about 400 times higher).

For the present work, the field blank filters were analyzed like sample filters, to obtain the ‘overall filter blanks’, which include the laboratory blanks, without any separation from other sources of blank (as carried out previously). Thus, the overall filter blanks reported here directly constitute the ‘blanks to be subtracted’ from the analytical signals (see Table 7, original data in Table S8). Despite a quite instable matrix, results from the various field blank filters showed a very good repeatability. The mean values of ‘blank to be subtracted’ obtained from all six filters were: Cd 10 ± 1 ng L^–1^, Pb 80 ± 7 ng L^–1^, Cu 0.36 ± 0.02 µg L^–1^. These values were used in the blank subtraction procedure applied for all the determinations.

Incidentally we can note that the present values are rather lower than those reported for previously prepared filters, i.e., Cd 42 ± 2 ng L^−1^, Pb 385 ± 27 ng L^−1^, 1.39 ± 0.02 µg L^−1^ [53], which means that the decontamination procedure of filters for the present work was remarkably improved with respect to the past.

Statistical analysis was carried out to better highlight possible differentiation between local and remote contributions to the metal contents. In particular, correlation analysis (CA), regression analysis (RA), and principal component analysis (PCA, see e.g., [134]) were used to study the possible relationships between metals (CA, RA, PCA) and between metals and samples (PCA). PCA was carried out on standardized data using as variables the contents of the three metals expressed either in mass fraction, or in atmospheric concentration, or in both measurement units. The UNISTAT Statistical Package Version 10, 2017 (Unistat Ltd., 9 South Close, Highgate, London N6 5UQ, UK) was used for all these analyses. The cross-validation of PCs was made by the SIMCA-P package, version 8.0, 1999 (Umetrics AB, Umeå, Sweden; today from Sartorius AG, Gottingen, Germany) in accordance with Wold’s procedure [163]. The PCA biplots, drawn with arrowheads at the ends of the vectors (see e.g., [134] (pp. 90–101)), were obtained by the S-PLUS package, version 6.2 Professional Edition (Lucent Technologies, Inc., Murray Hill, NJ, USA, 2003; from 2010 TIBCO Software Inc., Palo Alto, CA, USA).

Accuracy of the voltammetric determinations of trace and ultra-trace metals is routinely tested in our laboratory in a variety of environmental certified reference materials [30,53,157,164,165,166,167,168]. In particular, tests carried out during this work have already been published elsewhere both for trace metals in urban particulate matter reference materials (NIST 1648 and 1648a) [53] and, for ultra-trace metals, in open ocean seawater certified reference material (NASS-6), the last to test accuracy even at very low metal concentrations [168]. From the quoted references it can be seen that the results of the systematic measurements carried out on reference materials show that recoveries are, with few exceptions—between 95% and 105%—always within the tolerance intervals, and then in good agreement, of certified reference values, thus highlighting a good accuracy of all measurements. Concerning detection limits and repeatability of measurements, the reader is referred to the original set up work [53].

## 4. Conclusions

Concentrations of trace metals Cd, Pb, and Cu have been determined for the first time in the atmospheric aerosol of Dome C (Concordia Station, East Antarctic plateau) in two sites—close to and far from the station, respectively. Results allowed us to discriminate between two series of data, the first (the background series) from samples collected in conditions upwind of the station, mainly from the distant site but occasionally also from the other site, and the second (the contamination series) again from both sites but in conditions downwind of the station, that is, under the more or less direct influence of the station itself.

From the first series we determined the background metal contents of the area, which, expressed both in mass fractions and in atmospheric concentrations, resulted: Cd 1.2 ± 0.2 µg g^−1^ (0.24 ± 0.13 pg m^−3^), Pb (here fixed as upper limit) 113 ± 13 µg g^−1^ (21 ± 8 pg m^−3^), and Cu 0.91 ± 0.48 mg g^−1^ (0.12 ± 0.07 ng m^−3^). These values, compared also with the only other data availed in the literature for plateau sites, i.e., the South Pole, are interpreted as baseline contents of the three metals in the aerosol of air masses circulating in Central Antarctica in the area of Dome C. A generalized anthropic source via long-range transport from other low latitude continents, is hypothesized. Possible natural contributions are also argued for Cd, from volcanic sources arising from Antarctic volcanoes, and for Cu, from mineral dusts, rich of copper, coming from the southern most areas of South America and/or from the Antarctic Peninsula, where there are many copper ores, also in rocks not covered by ice.

The second series of data showed much higher values, with the following statistics: Cd 6.8 ± 1.3 µg g^−1^ (range 5.2–8.4 µg g^−1^) and 1.7 ± 1.1 pg m^−3^ (range 0.7–3.1 pg m^−3^), Pb 270 ± 180 µg g^−1^ (range 140–470 µg g^−1^), and 52 ± 9 pg m^−3^ (range 44–62 pg m^−3^), Cu 9.8 ± 8.9 mg g^−1^ (range 3.9–20 mg g^−1^), and 1.49 ± 0.94 ng m^−3^ (range 0.36–2.1 ng m^−3^). Thus, the metal contents increased by factors of 6–7 for Cd, 2.4–2.5 for Pb, and 11–12 for Cu. These increments resulted—with the exception of Cu for which the value was much higher—in the same proportion of the metal contents in the special diesel fuel (SAB) used at the station, clearly proving the local anthropic impact of the station as the main source of metal excesses in the contamination sample series. As for the much higher copper values with respect to the fuel ratios with Cd and Pb, we hypothesized that this could be the results of particularly intense atmospheric events in the Antarctic Peninsula and/or in southern South America.

We recognize that much more data is required to confirm the speculations, argumentations, and hypotheses formulated here on the interpretation of the results. In particular, the availability of samples with a much higher temporal resolution, and the determination of many other trace metals (with the isotopic ratios) and major ions, could together contribute to better characterize the chemical composition of the aerosol, to discriminate between natural/anthropic sources and local/remote origins, and to evaluate a quantitative source apportionment.

Chemical fractionation of metals obtained by sequential extraction of aerosol samples could also help in data interpretation in terms of sources and origins [53,115]. Three main fractions have been identified and related to different chemical compounds: (i) the water-soluble fraction, which contains marine salts and ions; (ii) the acid-extractable fraction, which contains mainly carbonates and sulphates ascribable to possible anthropogenic origin; and (iii) the inert fraction, constituted of oxides and silicates representing the possible contribution of the crustal dust.

The air–snow relationship is another aspect that should be considered in the future in order to determine metal depositional fluxes from the aerosol to the snow and to give elements for reconstructing past environmental changes from ice-core data concerning trace metals.

No data on trace metal concentrations are available until now at Dome C, so this study can be considered a starting point for future research programmes.

## Figures and Tables

**Table 1:** Cd, Pb, and Cu in Antarctic aerosol expressed as metal mass fraction and metal atmospheric concentration (±standard deviations from 3–6 measurements). Dome C, Concordia Station, austral summer 2005–2006. Atmospheric concentrations refer to standard air (298 K, 760 mmHg).

Sampling Point	Sample No.	Mass Fraction (±SD)				Atmospheric Concentration (±SD)		
		Cd (µg g^−1^)	Pb (mg g^−1^)	Cu (mg g^−1^)		Cd (pg m^−3^)	Pb (pg m^−3^)	Cu (ng m^−3^)
Concordia	1	7.0 ± 1.2	0.14 ± 0.02	5.4 ± 0.9		3.1 ± 0.4	62 ± 7	2.4 ± 0.3
	2	1.4 ± 0.4	0.11 ± 0.01	3.9 ± 0.6		0.40 ± 0.13	31 ± 3	1.1 ± 0.2
	3	5.2 ± 0.5	0.11 ± 0.01	0.92 ± 0.06		2.0 ± 0.2	44 ± 6	0.36 ± 0.04
Astrophysics T. 1	1	6.6 ± 1.6	0.47 ± 0.06	20 ± 2		0.70 ± 0.18	50 ± 7	2.1 ± 0.3
	2	1.3 ± 0.4	0.12 ± 0.01	1.06 ± 0.14		0.25 ± 0.09	23 ± 2	0.20 ± 0.03
	3	1.0 ± 0.5	0.13 ± 0.02	1.53 ± 0.12		0.09 ± 0.04	12 ± 1	0.14 ± 0.02
Astrophysics T. 2	1	8.4 ± 1.0	0.20 ± 0.03	0.88 ± 0.28		0.98 ± 0.14	24 ± 4	0.10 ± 0.03
	2	1.3 ± 0.4	0.10 ± 0.01	0.17 ± 0.03		0.20 ± 0.06	15 ± 2	0.027 ± 0.004

**Table 2:** Comparison of present results with other Antarctic data.

*Area* Station/site	Period	Metal Atmospheric Concentration, pg m^−3^			Reference
		Cd Average ± SD (min-max)	Pb Average ± SD (min-max)	Cu Average ± SD (min-max)	
*Antarctic Plateau*					
Dome C	Summer 2005–2006	0.24 ± 0.13 ^a^ (0.09–3.1)	21 ± 8 ^a^ (12–62)	120 ± 70 ^a^ (27–2400)	This work
South Pole	Summer 1970–1971	-	630 ± 300 (<190–1200)	36 ± 19 (25–64)	[31]
South Pole	Summer 1974–1975	<15	-	29 ± 17	[32]
South Pole	Summer 1974–1975	≤18	27 ± 10 (W) ^b^ 76 ± 40 (N) ^b^	29 ± 17	[33]
South Pole	Summers 1971–1978	49 ± 38	-	59 ± 47	[34]
	Winters 1975–1976	<200	-	79 ± 16	
South Pole	Summers 1979–1983	110 ± 60	-	190 ± 130	[35]
	Winters 1979–1983	50 ± 40	-	130 ± 80	
South Pole	November 2000–January 2001	-	<32 ^c^	-	[36]
South Pole	November 2000–January 2001	-	30 ± 10	-	[37]
South Pole	November–December 2003	-	180 ± 40	-	[37]
*Antarctic Peninsula*					
Spaatz Island Plateau site Rothera	Summer 1979–1980	3.5 ± 0.8 3.1 ± 2.1 5.4 ± 0.7	226 ± 35 79 ± 24 153 ± 60	- - -	[119]
Beethoven Peninsula	Summer 1982–1983	<1.1	<8.5	11.3 ± 6.2	[120]
Gipps Ice Rice	Summer 1984–1985	0.06 ± 0.1	4.7 ± 0.8	1.0 ± 1.0	[121]
Comandante Ferraz	Summers1985–1988	-	800 ^d^	690 ^d^	[14]
	Winters 1985–1988	-	830 ^d^	1200 ^d^	
	Overall 1985–1988	-	830 ^d^	990 ^d^	
Comandante Ferraz	Summers1985–1993		1200 ^d^	3450 ^d^	[15]
	Winters 1985–1993	-	970 ^d^	2740 ^d^	
	Overall 1985–1993	-	1060 ^d^	3010 ^d^	
King Sejong	January 2000–December 2001	1.3 ± 3 (0.07–16.7)	41 ± 54.5 (0.71–232)	143 ± 471 (2.0–2413)	[49]
*Antarctic Coast or Southern Ocean*					
Ekström Ice Shelf	April, May, December 1983	-	17 ± 9	-	[122]
Georg von Neumayer	Febuary 1983–December 1984	-	11 ± 1.5	-	[123]
Antarctic Ocean (Atlantic) includes Georg von Neumayer and Filchner Ice Shelf	Summer 1989–1990	26 ± 18	328 ± 269	163 ± 85	[124]
Antarctic Ocean	Summer 1986–1987	20	1100	2300	[125]
Antarctic Ocean	1989–1990	105	269	-	[126]
Southern Ocean	Summer 1999–2000	40	230	700	[127]
Southern Ocean coastal	Summer 2010–2011	4 (0–20)	-	-	[128]
Coastal East Antarctica	Summer 2010–2011	17(0–50)	-	-	[128]
Southern Ocean	Summer 2011–2012	17 ± 29	127 ± 98	957 ± 638	[129]
McMurdo Hut Point (downwind)	Summer 1995–1996 Summer 1996–1997 Total summers 1995–1997	- - -	1244 ± 2513 389 ± 541 851 ± 1915	194 ± 260 183 ± 104 189 ± 202	[28]
McMurdo Radar Sat (upwind)	Summer 1995–1996 Summer 1996–1997 Total summers 1995–1997	- - -	620 ± 1048 300 ± 438 470 ± 827	243 ± 304 152 ± 141 200 ± 244	[28]
Maitri	Summer 1990	-	306 ± 68	704 ± 339	[130]
Zhongshan Station	March 1998–Febuary 1999	70	559	1280	[131]
Zhongshan Station	March 1998–November 1999	47	431	778	[132]
Prydz Bay	Summer 1999–2000	30	80	460	[127]
Mario Zucchelli (formerly Terra Nova Bay) Faraglione Camp	Summer 2000–2001	-	16.7 (9.7–38.7)	422 (86–641)	[45]
	Summer 2001–2002	-	15.0 (6.8–48.7)	394 (121–1102)	[45]
	Summer 2010–2011	-	22.5 ^e^	32.5 ^e^	[47]
	Summers 1988–2002	-	10 ± 3	-	[133]
	Summer 2000–2001	3.4 ± 2.2 ^f^ (0.55–6.3)	24 ± 17 ^f^ (8.7–48)	266 ± 103 ^f^ (72–365)	[30]
	Summer 2000–2001	0.14–19 ^g^	17–36 ^g^	177–436 ^g^	[53]
	Summer 2000–2001	9.5 ± 13.1 ^g^ (0.93–39)	33 ± 16 ^g^ (17–60)	340 ± 150 ^g^ (88–480)	[115]
Larsemann Hills Antarctic Coast	Summer 2009–2010	-	260 ± 180	2190 ± 530	[43]
Larsemann Hills Southern Ocean	Summer 2009–2010	-	580 ± 190	9020 ± 5800	[43]

^a^ Background values; ^b^ W = Watman filter, N = Nuclepore filter; ^c^ Lead quantified only in 15 samples (above detection limit) out of 53 total: 32 ± 8 pg m^−3^; ^d^ Fine (*d*_p_ < 2.0 µm) plus coarse (2.0 < *d*_p_ < 15.0 µm) modes of Antarctic aerosol; ^e^ Lower limits, data does not include the size fraction < 0.49 µm; ^f^ Total extractable; ^g^ Total.

**Table 3:** Correlation coefficients (r) and significance (*p*-value) between metal contents expressed in both measurement units (mass fraction and atmospheric concentration) for the whole series of samples. One-tailed *t*-test is used since the interest is for r > 0.

Metals	Mass Fraction			Atmospheric Concentration	
	*r*	*p*-Value (*)		*r*	*p*-Value (*)
Cd-Pb	0.50	0.10		0.83	0.006
Cd-Cu	0.38	0.17		0.57	0.071
Pb-Cu	0.93	0.0004		0.87	0.003

(*) One-tail probability.

**Table 4:** Correlation coefficients (*r*) and significance (*p*-value) between metal contents in samples of the background series (CS-2, AT1-2, AT1-3, AT2-2) and in those affected by the station activities (CS-1, CS-3, AT1-1, AT2-1). One-tailed *t*-test is used since the interest is for *r* > 0.

Metals	Background Samples						Contamination Samples				
	Mass Fraction			Atmospheric Conc.			Mass Fraction			Atmospheric Conc.	
	*r*	*p*-Value *		*r*	*p*-Value*		*r*	*p*-Value *		*r*	*p*-Value *
Cd-Pb	−0.69	0.16		0.97	0.016		0.12	0.44		0.65	0.19
Cd-Cu	0.33	0.34		0.86	0.072		−0.084	0.46		0.34	0.33
Pb-Cu	0.17	0.42		0.88	0.062		0.94	0.032		0.87	0.064

(*) One-tail probability.

**Table 5:** Comparison of regression coefficients obtained from full and separate datasets. Mass fractions in µg g^−1^, atmospheric concentrations in pg m^−3^, regression coefficients in µg_metal A_/µg_metal B_ or pg_metal A_/pg_metal B_, respectively.

Regression	Total Dataset		Separate Datasets	
	from Mass Fraction *from atmospheric conc.*		Background Samples from Mass Fraction *from atmospheric conc.*	Contamination Samples from Mass Fraction *from atmospheric conc.*
Pb vs. Cd	14		64	9.4
	*20*		*−58*	*15*
Cu vs. Cd	510		3300	370
	*810*		*3000*	*−570*
Cu vs. Pb	47		51	65
	*49*		*18*	*52*

**Table 6:** Metal contents and metal ratios in the fuels used at Concordia Station. Data in mass fraction.

Fuel	Metal Contents µg _metal_/kg _fuel_				Metal Ratios µg _metal A_/µg _metal B_		
	Cd	Pb	Cu		Pb/Cd	Cu/Cd	Cu/Pb
SAB diesel fuel	0.7 ± 0.2	10 ± 1	70 ± 7		14 ± 4	100 ± 30	7 ± 1
Unleaded gasoline	1.0 ± 0.2	65 ± 3	101 ± 9		65 ± 13	101 ± 22	1.6 ± 0.2
Jet A-1 fuel	7 ± 3	80 ± 2	192 *±* 17		11 ± 5	27 ± 12	2.4 ± 0.2

**Table 7:** Blank values obtained analyzing the field blank filters for Cd, Pb, and Cu. Conditions: 1/8 filter, 100 mL final solution after digestion. Blanks include laboratory blanks (a sort of filter blanks gross of laboratory blanks); thus, they represent the blanks to be subtracted.

Field Blank Filter	Metal Concentrations (Mean ± SD)		
	Cd (ng L^−1^)	Pb (ng L^−1^)	Cu (µg L^−1^)
Concordia, blank 1	10.0 ± 0.3 (*n* = 3)	84 ± 5 (*n* = 4)	0.39 ± 0.02 (*n* = 5)
Concordia, blank 2	9.4 ± 0.8 (*n* = 4)	82 ± 8 (*n* = 4)	0.34 ± 0.03 (*n* = 4)
Astrophysics T. 1, blank 1	10.1 ± 1.0 (*n* = 3)	75 ± 7 (*n* = 3)	0.34 ± 0.02 (*n* = 3)
Astrophysics T. 1, blank 2	9.4 ± 1.8 (*n* = 3)	80 ± 9 (*n* = 3)	0.40 ± 0.03 (*n* = 3)
Astrophysics T. 2, blank 1	10.7 ± 0.3 (*n* = 3)	82 ± 4 (*n* = 3)	0.34 ± 0.02 (*n* = 4)
Astrophysics T. 2, blank 2	10.0 ± 1.8 (*n* = 3)	76 ± 9 (*n* = 4)	0.37 ± 0.03 (*n* = 3)
Weighted mean ± SD i.e., blank to be subtracted	10 ± 1 (*n* = 19)	80 ± 7 (*n* = 21)	0.36 ± 0.02 (*n* = 22)

## Data Availability

Original data are reported in Supplementary Materials (Section S9) and they are available from the authors.

## Supplementary Materials

The following are available online, Section S1: Backward trajectories (Figure S1: NOAA Hysplit five-day air backward trajectories run for arrival heights of 100 m, 500 m, and 1000 m a.g.l. at 12 a.m. of each day recorded at Dome C during the summer 2005–2006); Section S2: Principal component analysis (Table S1: PCA results from mass fraction data (Unistat package); Table S2: Cross-validation (Significance) and PCA results from SIMCA package: mass fraction data; Figure S2: PCA biplots from mass fraction data on the plane of the first two PCs; Table S3: PCA results from atmospheric concentration data (Unistat package); Table S4: Cross-validation (Significance) and PCA results from SIMCA package: atmospheric concentration data; Figure S3: PCA biplots from atmospheric concentration data on the plane of the first two PCs; Table S5: PCA results from mass fraction data and atmospheric concentration data (Unistat package); Table S6: Cross-validation (Significance) and PCA results from SIMCA package: mass fraction plus atmospheric concentration data; Figure S4: PCA biplots from mass fraction data and atmospheric concentration data on the planes of components PC2 vs. PC1 and PC3 vs. PC1); Section S3: Regression analysis for the whole dataset and the two subsets of background and contamination data (Figure S5: Bivariate scatter plots of metal contents in the aerosol (in mass fractions and atmospheric concentrations), with in evidence the two subsets representative of the background and contamination samples); Section S4: Map of the sampling site (Figure S6: Technical map of the sampling area, with locations of aerosol sampling points at Concordia Station, Astrophysics Tent 1 and Astrophysics Tent 2); Section S5: Images from sampling activities (Figure S7: Sampler location at sampling point Concordia Station, the site of astrophysics tent with the location of the two samplers in this site, and satellite images); Section S6: Decontamination of filters (Table S7: Results of a filter decontamination procedure); Section S7: Images from the treatment and analysis of filters in the Antarctic and Italian laboratories (Figure S8: Filter conditioning and weighing in the clean laboratory of Concordia Station, Antarctica, and filter cutting and voltammetric analysis in our clean chemistry laboratory, Italy); Section S8: Potential-time waveform applied in voltammetric analysis (Figure S9: Potential-time waveform and current sampling scheme in square-wave voltammetry); Section S9: Original data (Table S8: Overall filter blanks, Table S9: Original results from sample analyses).

## Sample Availability

Aliquots of samples are available from the authors.
