# Erratum: Illuminati et al. Determination of Cd, Pb, and Cu in the Atmospheric Aerosol of Central East Antarctica at Dome C (Concordia Station). *Molecules* 2021, *26*, 1997

**DOI:** 10.3390/molecules26175254

**Published:** 2021-08-30

**Authors:** Silvia Illuminati, Anna Annibaldi, Cristina Truzzi, Caterina Mantini, Eleonora Conca, Mery Malandrino, Giada Giglione, Matteo Fanelli, Giuseppe Scarponi

**Affiliations:** 1 Dipartimento di Scienze della Vita e dell’Ambiente, Università Politecnica delle Marche, Via Brecce Bianche, 60131 Ancona, Italy; s.illuminati@univpm.it (S.I.); caterina.mantini@libero.it (C.M.); g.giglione@pm.univpm.it (G.G.); matteo.fanelli@virgilio.it (M.F.); g.scarponi@univpm.it (G.S.); 2 Department of Analytical Chemistry, University of Torino, Via Giuria 5, 10125 Torino, Italy; eleonora.conca@unito.it (E.C.); mery.malandrino@unito.it (M.M.)

The authors wish to make the following corrections to the paper [1]: Two *p*-values referred to correlations in Section 2.4 are not correct due to oversight. 

Original:

The most significant correlation (*p*-value < 0.05) is between lead and copper, both in mass fraction and in the atmospheric concentration. Cadmium and lead show a high correlation (at the limits of significance) only for the atmospheric concentration (*p* = 0.06).

We would thus like to make following changes to the paper [1], as follows: 

The most significant correlation (*p*-value < 0.005) is between lead and copper, both in mass fraction and in the atmospheric concentration. Cadmium and lead show a high correlation only for the atmospheric concentration (*p* = 0.006).

We apologize for this unintended mistake, which, however, does not affect the results of this manuscript and the conclusions drawn from them.
