# Supplementary material for: Determination of Cd, Pb, and Cu in the Atmospheric Aerosol of Central East Antarctica at Dome C (Concordia Station)
Source: Molecules. 2021 Apr 1;26(7):1997. doi: 10.3390/molecules26071997 (PMC8036987; doi:10.3390/molecules26071997)
Supplement: Supplementary file 1 [file molecules-26-01997-s001.pdf]

Supplementary Materials

Determination of Cd, Pb, and Cu in the atmospheric aerosol of Central East Antarctica at Dome C (Concordia Station)

Silvia Illuminati[1], Anna Annibaldi[1*], Cristina Truzzi[1*], Caterina Mantini[1], Eleonora Conca[2], Mery Malandrino[2], Matteo Fanelli[1], Giada Giglione[1], and Giuseppe Scarponi[1]

[1] Dipartimento di Scienze della Vita e dell'Ambiente, Università Politecnica delle Marche, Via Brecce Bianche, 60131 Ancona, Italy; s.illuminati@univpm.it (S.I.); a.annibaldi@univpm.it (A.A); c.truzzi@univpm.it (C.T.); caterina.mantini@libero.it (C.M.); m.fanelli@univpm.it (M.F.); g.giglione@univpm.it (G.G.); g.scarponi@univpm.it (G.S.)

[2] Department of Analytical Chemistry, University of Torino, Via Giuria 5, 10125 Torino, Italy; eleonora.conca@unito.it (E.C.); mery.malandrino@unito.it (M.M.)

[*] Correspondence: a.annibaldi@univpm.it (A.A.); c.truzzi@univpm.it (C.T.); Tel.: +39-071-2204981 (A.A., C.T., S.I.)

Contents

From "Results and Discussion"

Section S1. Backward trajectories (Figure S1)

Section S2. Principal component analysis (Tables S1-S2, Figure S2; Tables S3-S4, Figure S3; Tables S5-S6, Figure S4)

Section S3. Regression analysis for the whole dataset and the two subsets of background and contamination data (Figure S5)

From "Materials and Methods"

Section S4. Map of the sampling site (Figure S6)

Section S5. Images from sampling activities (Figure S7)

Section S6. Decontamination of filters (Table S7)

Section S7. Images from the treatment and analysis of filters in the Antarctic and Italian laboratories (Figure S8)

Section S8. Potential-time waveform applied in voltammetric analysis (Figure S9)

Section S9. Original data (Tables S8-S9)

Section S1. Backward trajectories

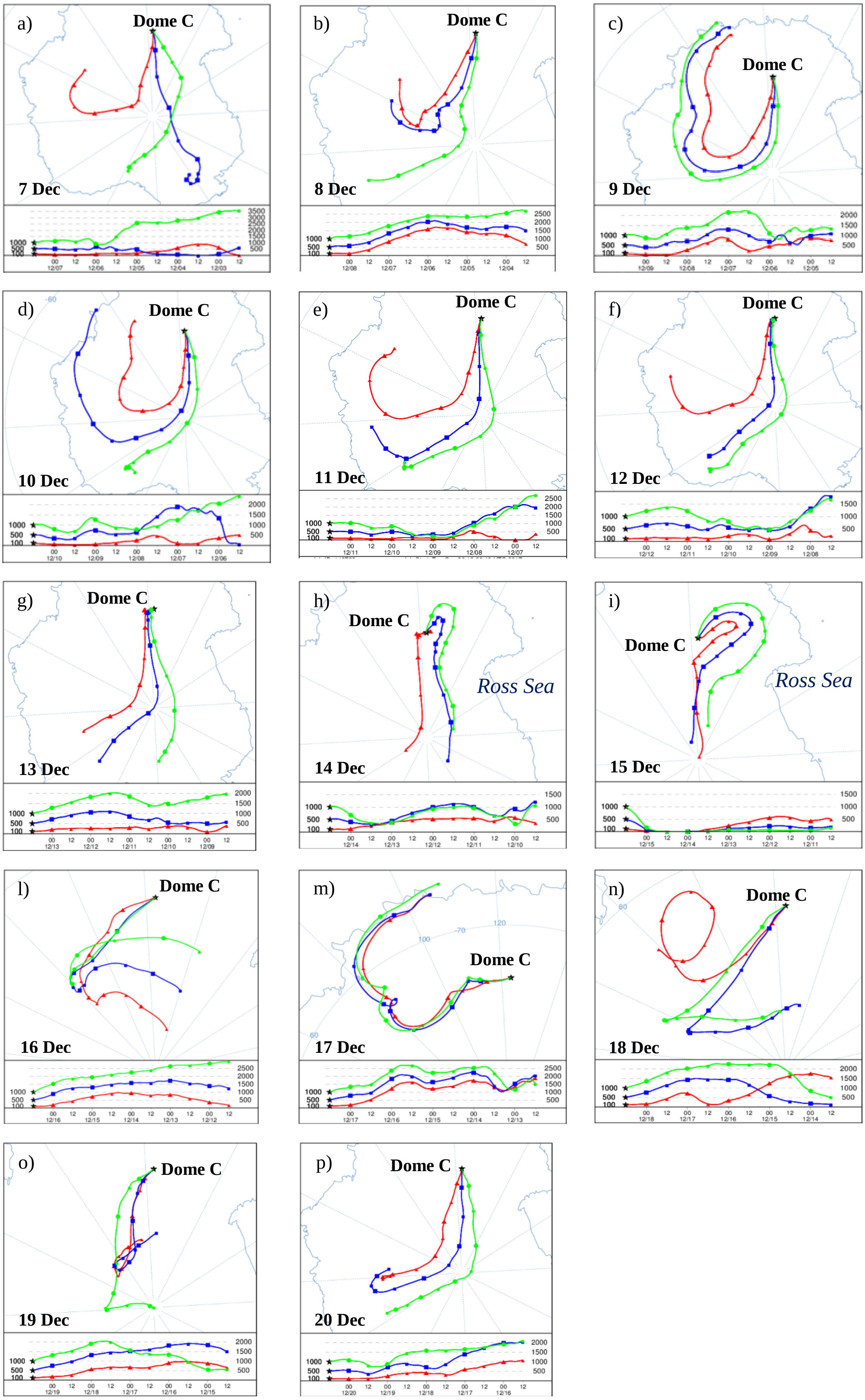

Figure S1. NOAA Hysplit five-day air backward trajectories run for arrival heights of 100 m (red), 500 m (blue) and 1000 m (green) a.g.l. at 12 a.m. of each day recorded at Dome C during the summer 2005-2006. (**a**) Period Dec 7-20, 2005. Continue.

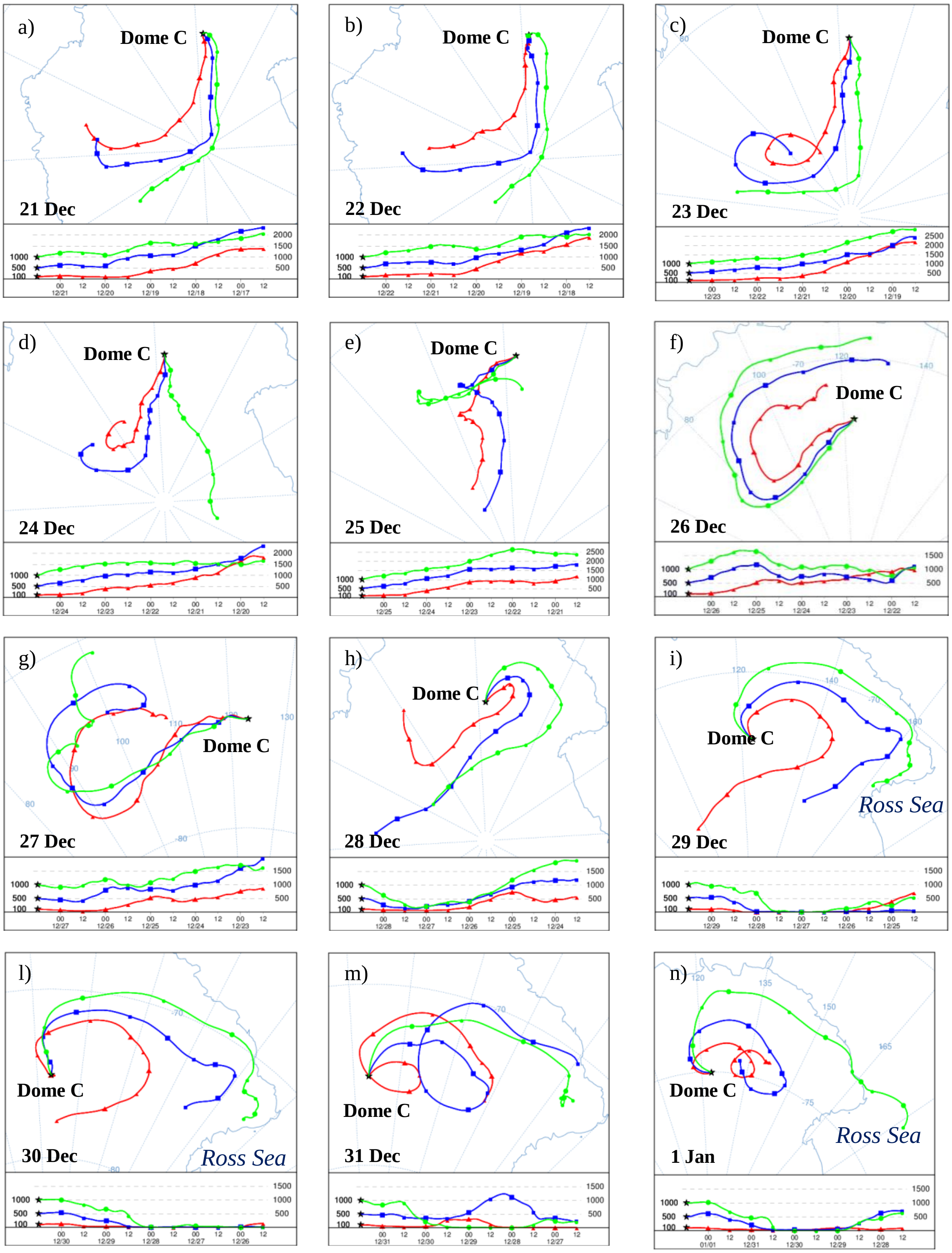

Figure S1. Continuation. (**b**) Period Dec 21, 2005, to Jan 1, 2006.

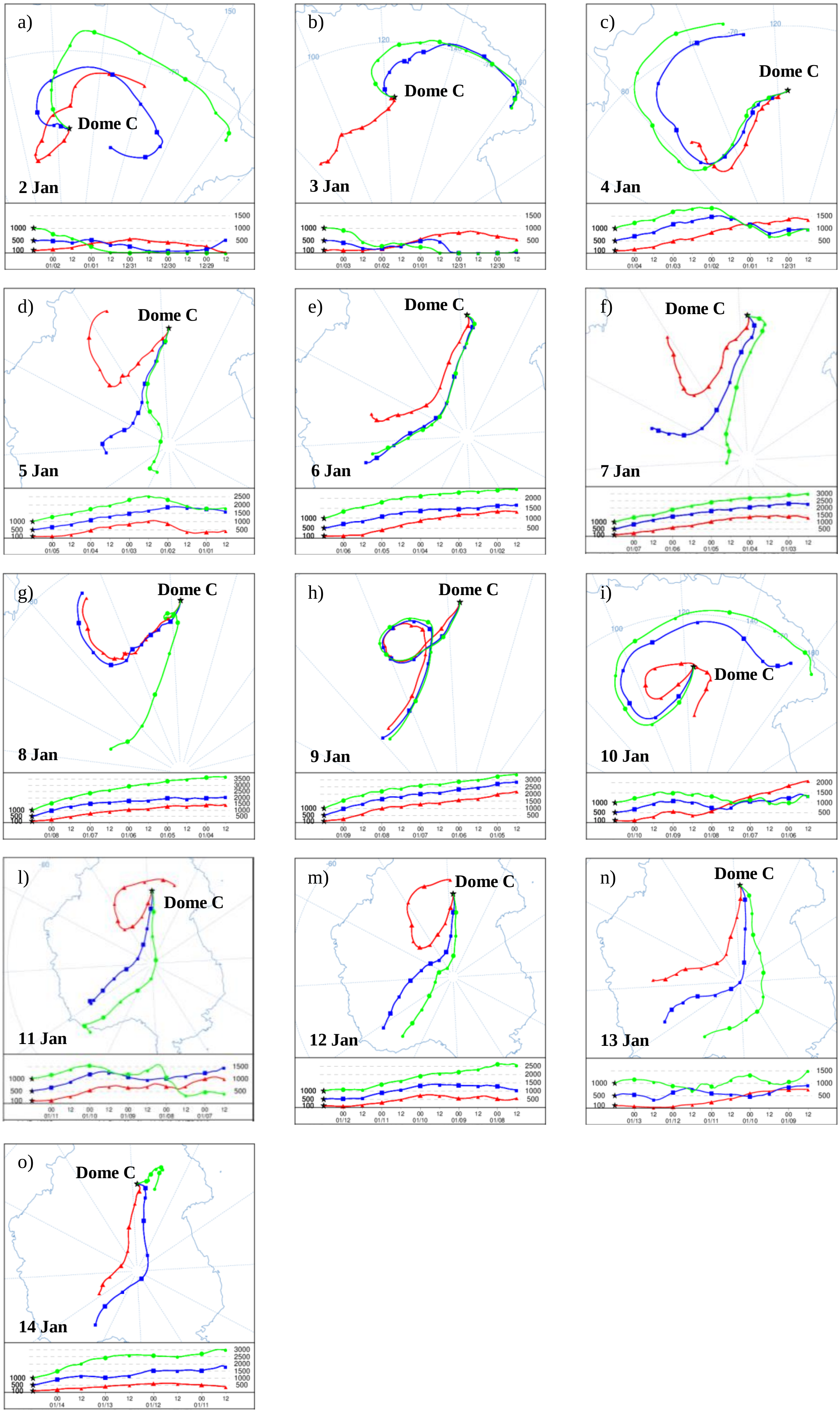

Figure S1. Continuation. (**c**) Period Jan 2-14, 2006.

Section S2. Principal component analysis

Principal Component Analysis (PCA) was used to study the possible relationships between samples (score plots), between metals (loading plots) and between samples and metals (PCA biplots). PCA was carried out on standardized data using as variables the contents of the three metals expressed either in mass fraction, or in atmospheric concentration, or in both measurement units. The UNISTAT Statistical Package Version 10, 2017 (Unistat Ltd., 9 South Close, Highgate, London N6 5UQ, UK) was used for all these analyses. The cross-validation of PCs was made by the SIMCA-P package, version 8.0, 1999 (Umetrics AB, Umeå, Sweden; today from Sartorius AG, Gottingen, Germany) in accordance with Wold's procedure ([163] Wold, 1978). The PCA biplots with arrowheads at the ends of the vectors, see e.g. [134] (pp. 90-110), were obtained by the S-PLUS package, version 6.2 Professional Edition (Lucent Technologies, Inc., Murray Hill, NJ, USA, 2003; from 2010 TIBCO Software Inc., Palo Alto, CA, USA).

S2.1. PCA from mass fraction data

Results obtained using mass fraction as expression of the metal contents are reported in Tables S1 and S2 and in Figure S2. The first two PCs are significant at the cross-validation criterion (Table S2), together they account for ~98% of the total variation (PC1 ~75%, PC2 ~23%), and are dicussed here. The first PC expresses the overal metal loading, while the second PC contrasts Cd on one hand with Pb and Cu on the other (Figure S2). The scatter plot of samples (see in particular Figure S2b) shows a clear separation between samples with low metal contents (AT1-2, AT1-3, AT2-2, CS2) and those with generally much higher values, but with particular reference to Cd (CS1, CS3, AT2-1), or to Pb and Cu (AT1-1).

S2.2. PCA from atmospheric concentration data

Results obtained using atmospheric concentrations as expression of the metal contents are reported in Tables S3 and S4 and in Figure S3. The first two PCs are significant at the cross-validation criterion (Table S4), together they account for ~98% of the total variation (PC1 ~84%, PC2 ~14%), and are dicussed here. The first PC expresses the overal metal loading, the second PC contrasts Cd on one hand with Cu on the other, while Pb has no influence on PC2 (Figure S3). The different structure of the loading plot with respect to the previous case of mass fraction data can be ascribed to the fact that in this case we analyse the metal contents referred to the atmospheric volume instead of to the mass of the particulate matter. Here the aerosol concentration in the atmosphere enter in the expression of the metal content, in addition to the mass fraction measured in the solid particulate matter. The scatter plot of samples (see in particular Figure S3b) shows again a clear separation between samples with low metal contents (AT1-2, AT1-3, AT2-2), where however the CS2 sample is not included as in the previous case, and those with generally much higher values, with particular reference to Cd (CS1, CS3, AT2-1), to Cu (AT1-1, CS-1, CS-2), or to Pb (CS-1, AT1-1, CS-3).

S2.3. PCA from mass fraction plus atmospheric concentration data

Here to distinguish between the two kinds of measurements units we used for mass fractions the symbols Cd.F, Pb.F, Cu.F, and for atmospheric concentrations Cd.C, Pb.C, Cu.C. Results obtained using both mass fractions and atmospheric concentrations as expression of the metal contents, are reported in Tables S5 and S6 and in Figure S4. Here the first three PCs are significant at the cross-validation criterion (Table S6), together they account for ~98% of the total variation (PC1 ~62%, PC2 ~26%, PC3 ~10%), and are dicussed here. The first PC expresses again the overal metal loading. The second PC contrasts Pb and Cu mass fracrions (Pb.F, Cu.F), on one hand, with Pb and Cd atmospheric concentrations (Pb.C, Cd.C), on the other (Figure S4b). The third PC contrasts Pb and Cd mass fracrions (Pb.F, Cd.F), on one hand, with Pb and Cu atmospheric concentrations (Pb.C, Cu.C), on the other (Figure S4d). The scatter plot of samples (see in particular Figure S4b,d) definitely confirms the separation of four samples with low metal contents (AT1-2, AT1-3, AT2-2, CS2) from those with generally much higher values, considering mass fractions and atmospheric concentrations together.

Table S1. PCA results from mass fraction data (Unistat package).

Variance Table

Component No	Eigenvalue	Cumulative Variance	Percent	Cumulative %
1	2.2478	2.2478	74.93	74.93
2	0.6961	2.9440	23.20	98.13
3	0.0560	3.0000	1.87	100.00

Eigenvectors

	Component 1	Component 2	Component 3
Cd	0.4491	0.8855	0.1193
Pb	0.6445	-0.2286	-0.7296
Cu	0.6188	-0.4045	0.6734

Principal Components

	Component 1	Component 2	Component 3
CS-1	0.3761	0.8393	0.4215
CS-2	-0.7330	-0.6180	0.2285
CS-3	-0.4597	0.6527	0.0718
AT1-1	3.3917	-0.7740	-0.0412
AT1-2	-0.9614	-0.4913	-0.1230
AT1-3	-0.9121	-0.6225	-0.1484
AT2-1	0.4673	1.4065	-0.3361
AT2-2	-1.1689	-0.3928	-0.0730

Table S2. Cross-validation (Significance) and PCA results from SIMCA package: mass fraction data.

Simca-P 8.0
Project Aerosol Dome C
Model M1
Data set Mass Fractions (Cd, Pb, Cu)
Type PC-X
NObs 8 NVarX 3 NVarY 0
Components:

A	R2X	R2X(cum)	Eigenvalues	Q2	Limit	Q2(cum)	Significance	Iterations
00	-	0.000	-	-	-	-	--	--
01	0.749	0.749	2.248	0.001	0.344	0.001	NS	8
02	0.232	0.981	0.696	0.754	0.429	0.754	R1	5
03	0.019	1.000	0.056	1.000	0.583	1.000	N3	1

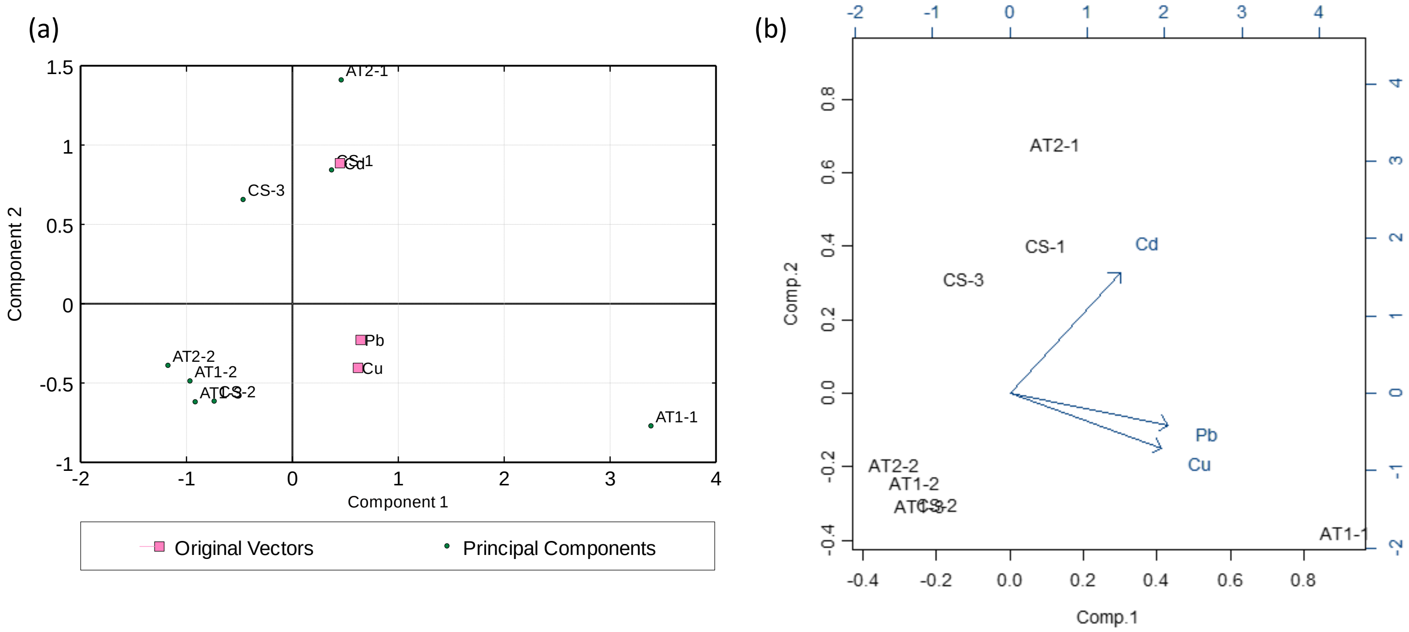

Figure S2. PCA biplots from mass fraction data on the plane of the first two PCs; (a) Unistat package and (b) S-Plus package (biplot version with directed line segments). Explained variances: PC1 ~75%, PC2 ~23%.

Table S3. PCA results from atmospheric concentration data (Unistat package).

Variance Table

Component No	Eigenvalue	Cumulative Variance	Percent	Cumulative %
1	2.5165	2.5165	83.88	83.88
2	0.4339	2.9504	14.46	98.35
3	0.0496	3.0000	1.65	100.00

Eigenvectors

	Component 1	Component 2	Component 3
Cd	0.5486	0.7357	0.3972
Pb	0.6205	-0.0398	-0.7832
Cu	0.5604	-0.6761	0.4783

Principal Components

	Component 1	Component 2	Component 3
CS-1	3.0665	0.2869	0.2991
CS-2	-0.1754	-0.5976	0.0085
CS-3	0.6737	1.0052	-0.3366
AT1-1	1.2301	-1.1384	-0.2190
AT1-2	-1.0597	-0.0480	-0.1439
AT1-3	-1.5626	-0.0919	0.2522
AT2-1	-0.7058	0.5265	0.0353
AT2-2	-1.4668	0.0574	0.1044

Table S4. Cross-validation (Significance) and PCA results from SIMCA package: atmospheric concentration data.

Simca-P 8.0
Project Aerosol Dome C
Model M2
Data set Atm Conc (Cd, Pb, Cu)
Type PC-X
NObs 8 NVarX 3 NVarY 0
Components:

A	R2X	R2X(cum)	Eigenvalues	Q2	Limit	Q2(cum)	Significance	Iterations
00	-	0.000	-	-	-	-	--	--
01	0.839	0.839	2.517	0.439	0.344	0.439	R1	6
02	0.145	0.983	0.434	0.552	0.429	0.749	R1	13
03	0.017	1.000	0.050	1.000	0.583	1.000	N3	1

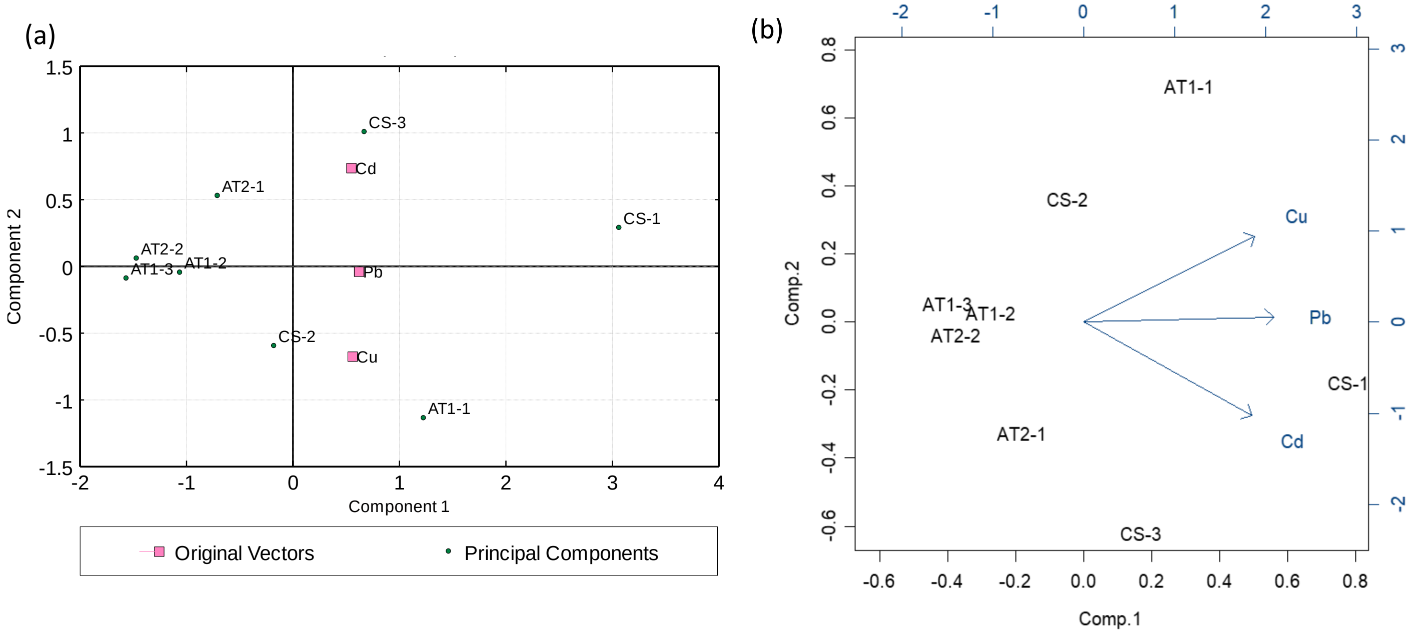

Figure S3. PCA biplots from atmospheric concentration data on the plane of the first two PCs; (a) Unistat package and (b) S-Plus package (biplot version with directed line segments). Explained variances: PC1 ~84%, PC2 ~14%. Note that the polarity of PC2 axis in S-Plus plot is reversed with respect to that in Unistat plot.

Table S5. PCA results from mass fraction data (Cd.F, Pb.F, Cu.F) and atmospheric concentration data (Cd.C, Pb.C, Cu.C) (Unistat package).

Variance Table

Component No	Eigenvalue	Cumulative Variance	Percent	Cumulative
1	3.7476	3.7476	62.46	62.46
2	1.5391	5.2868	25.65	88.11
3	0.6185	5.9053	10.31	98.42
4	0.0730	5.9783	1.22	99.64
5	0.0199	5.9982	0.33	99.97
6	0.0018	6.0000	0.03	100.00

Eigenvectors

	Component 1	Component 2	Component 3	Component 4	Component 5	Component 6
Cd.F	0.3916	0.1881	0.7637	-0.3394	-0.2675	0.2030
Pb.F	0.3615	-0.5426	0.2911	0.1809	0.2834	-0.6141
Cu.F	0.4055	-0.4881	-0.1455	0.1933	0.1369	0.7211
Cd.C	0.3346	0.6075	-0.0002	0.2007	0.6908	0.0381
Pb.C	0.4741	0.2508	-0.2334	0.5255	-0.5934	-0.1721
Cu.C	0.4635	-0.0037	-0.5062	-0.7059	-0.0066	-0.1748

Principal Components

	Component 1	Component 2	Component 3	Component 4	Component 5	Component 6
CS-1	2.5875	1.8657	-0.5970	-0.2414	0.0899	-0.0193
CS-2	-0.6107	-0.2130	-0.9216	-0.1853	-0.2363	0.0389
CS-3	0.1820	1.3410	0.3026	0.5441	-0.0151	0.0299
AT1-1	3.1668	-2.2165	0.0716	0.1201	0.0187	0.0037
AT1-2	-1.4654	-0.2486	-0.2792	0.1553	-0.0873	-0.0900
AT1-3	-1.8210	-0.5899	-0.1630	-0.0965	0.2349	0.0010
AT2-1	-0.1361	0.2806	1.7055	-0.2717	-0.0809	-0.0031
AT2-2	-1.9030	-0.2195	-0.1189	-0.0246	0.0760	0.0389

Table S6. Cross-validation (Significance) and PCA results from SIMCA package: mass fraction plus atmospheric concentration data.

Simca-P 8.0
Project Aerosol Dome C
Model M3
Data set Mass + Atm Conc (Cd, Pb, Cu)
Type PC-X
NObs 8 NVarX 6 NVarY 0
Components:

A	R2X	R2X(cum)	Eigenvalues	Q2	Limit	Q2(cum)	Significance	Iterations
00	-	0.000	-	-	-	-	--	--
01	0.625	0.625	3.748	-0.119	0.250	0.000	NS	10
02	0.257	0.881	1.539	0.471	0.286	0.471	R1	9
03	0.103	0.984	0.619	0.429	0.333	0.698	R1	8
04	0.012	0.996	0.073	0.540	0.400	0.861	N3	8
05	0.003	1.000	0.020	0.879	0.500	0.983	N3	6
06	0.000	1.000	0.002	1.000	0.667	1.000	N3	1

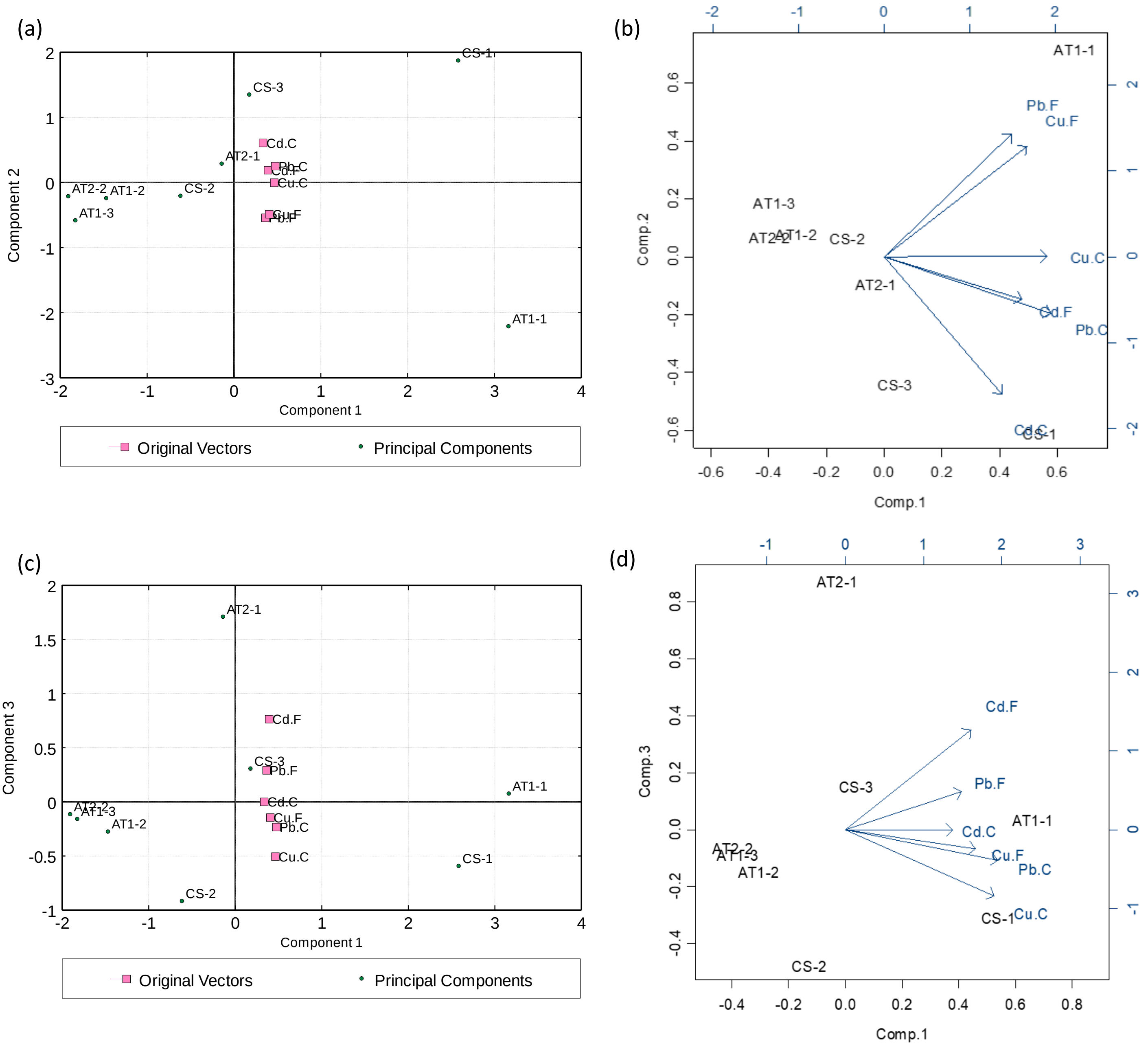

Figure S4. PCA biplots from mass fraction data (Cd.F, Pb.F, Cu.F) and atmospheric concentration data (Cd.C, Pb.C, Cu.C) on the planes of components PC2 vs PC1 (a and b) and PC3 vs PC1 (c and d), respectively; (a, c) Unistat package and (b, d) S-Plus package (biplot versions with directed line segments). Explained variances: PC1 ~62%, PC2 ~26%, PC3 ~10%. Note that the polarity of PC2 axis in S-Plus plot is reversed with respect to that in Unistat plot.

Section S3. Regression analysis for the whole dataset and the two subsets of background and contamination data

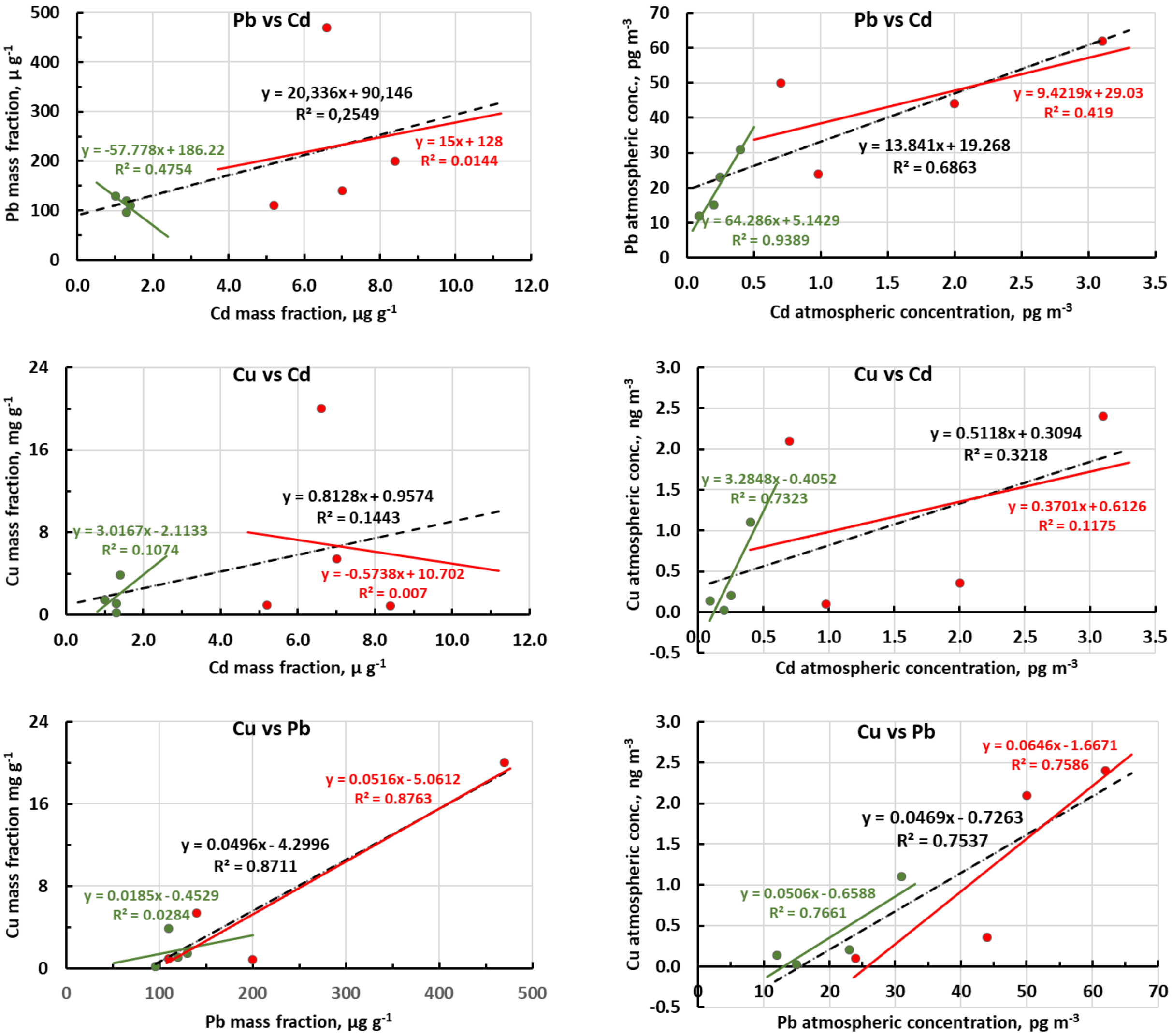

Figure S5. Bivariate scatter plots of metal contents in the aerosol (in mass fractions and atmospheric concentrations), with in evidence the two subsets representative of the background (green) and contamination (red) samples. Dome C austral summer 2005-06.

Section S4. Map of the sampling site

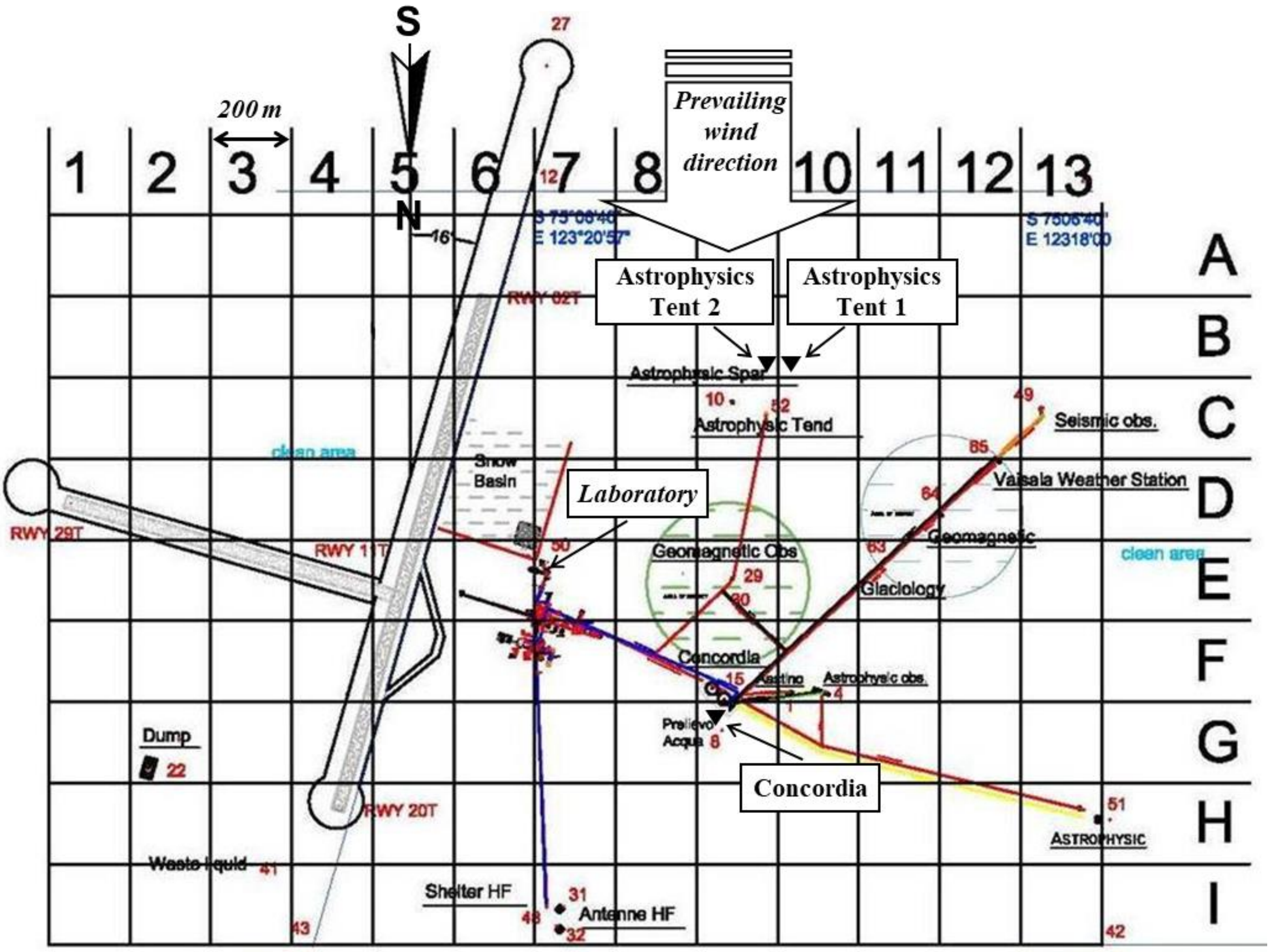

Figure S6. Technical map of the sampling area, with locations of aerosol sampling points at Concordia Station (Concordia, CS), Astrophysics Tent 1 (AT1) and Astrophysics Tent 2 (AT2).

Section S5. Images from sampling activities

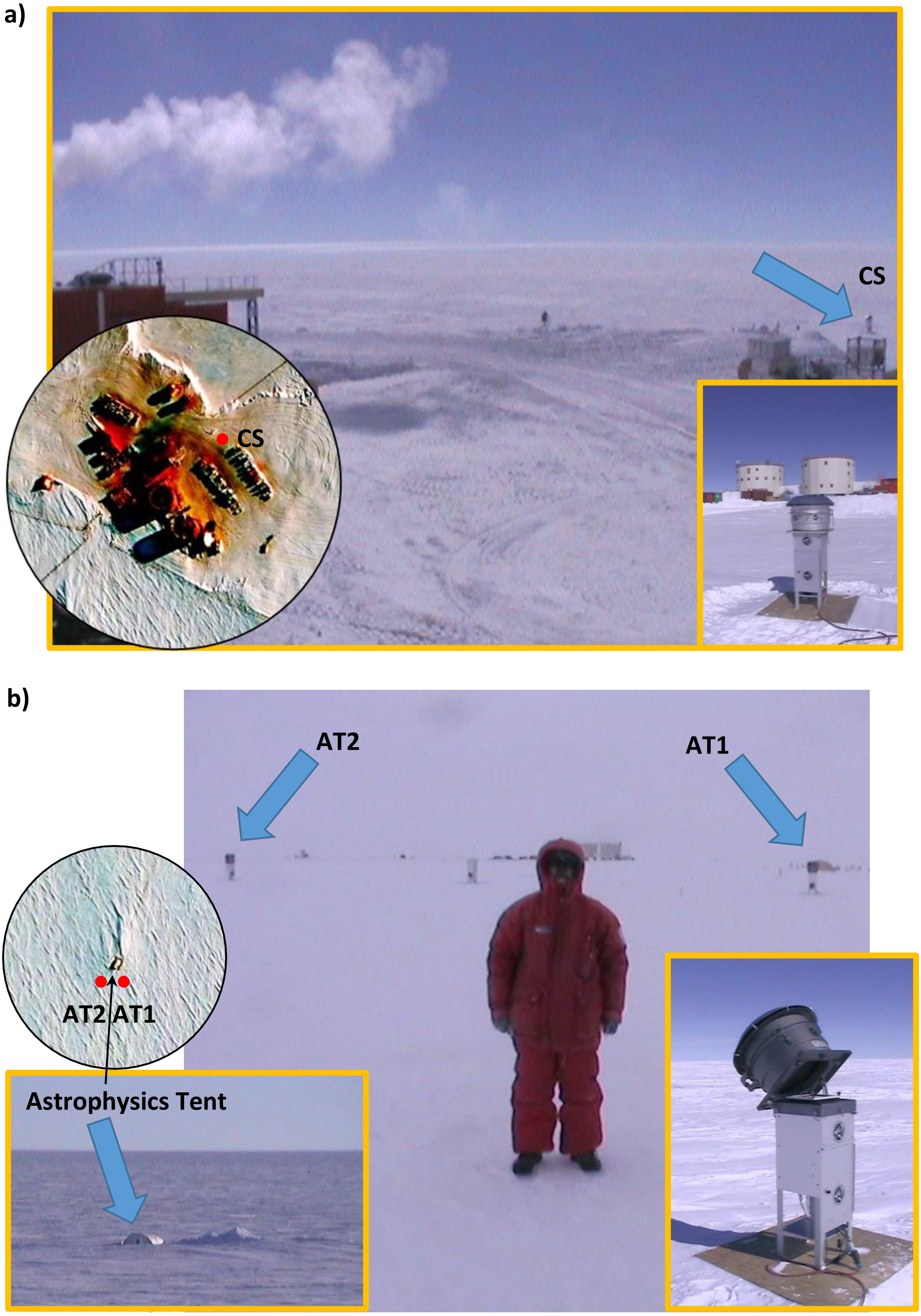

Figure S7. (**a**) Sampler location at sampling point Concordia Station (Concordia, CS). The arrow points to the sampler in the large photo. Note the direction of the smoke from the Station towards the sampler (enlarged in the insert). (**b**)The site of Astrophysics Tent (left), the location of the two samplers (AT1, AT2) in this site (centre), and one sampler in the foreground (right). Satellite images on the circles (from Figure 8).

Section S6. Decontamination of filters

Table S7. Results of a filter decontamination procedure. Standard deviations from three measurements at least.

Date	Treatment	Metal concentrations (mean ±SD)		
		Cd (ng l^{-1})	Pb (ng l^{-1})	Cu (µg l^{-1})
28 May 2005	24 h Ultrapure water	0.20 ±0.05	5.3 ±0.2	11.2 ±0.5
30 May 2005	24 h Ultrapure HCl 1+1000	790 ±8	160 ±8	63 ±3
01 June 2005	24 h Ultrapure water	0.51 ±0.06	3.5 ±0.3	nd
02 June 2005	24 h Ultrapure HCl 1+1000	7.4 ±0.2	4.2 ±0.4	7.4 ±0.3
03 June 2005	24 h Ultrapure water	nd	nd	nd
04 June 2005	24 h Ultrapure HCl 1+1000	nd	nd	nd

nd = signal non detected.

Section S7. Images from the treatment and analysis of filters in the Antarctic and Italian laboratories

a)

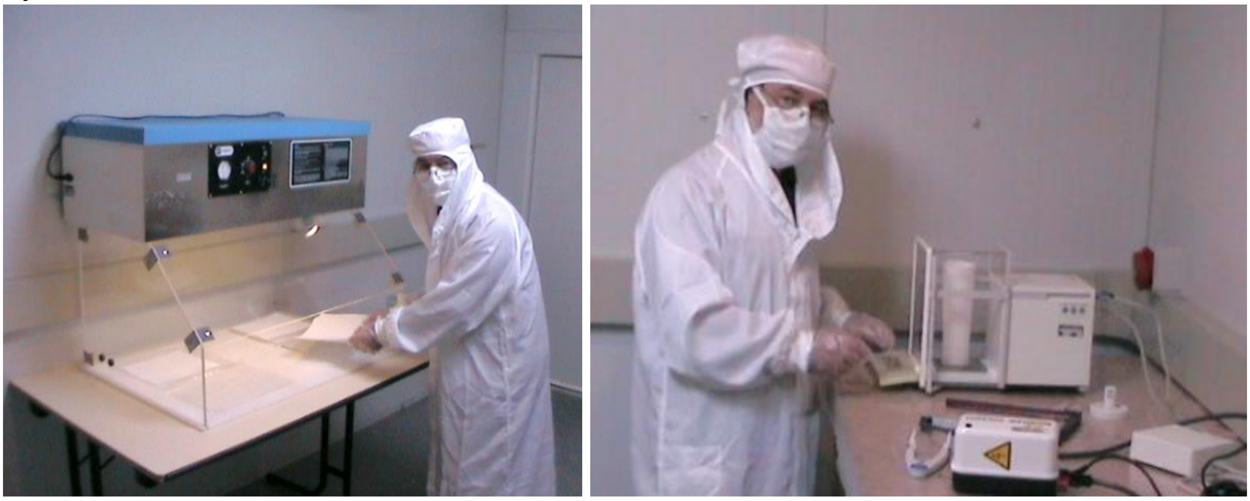

b)

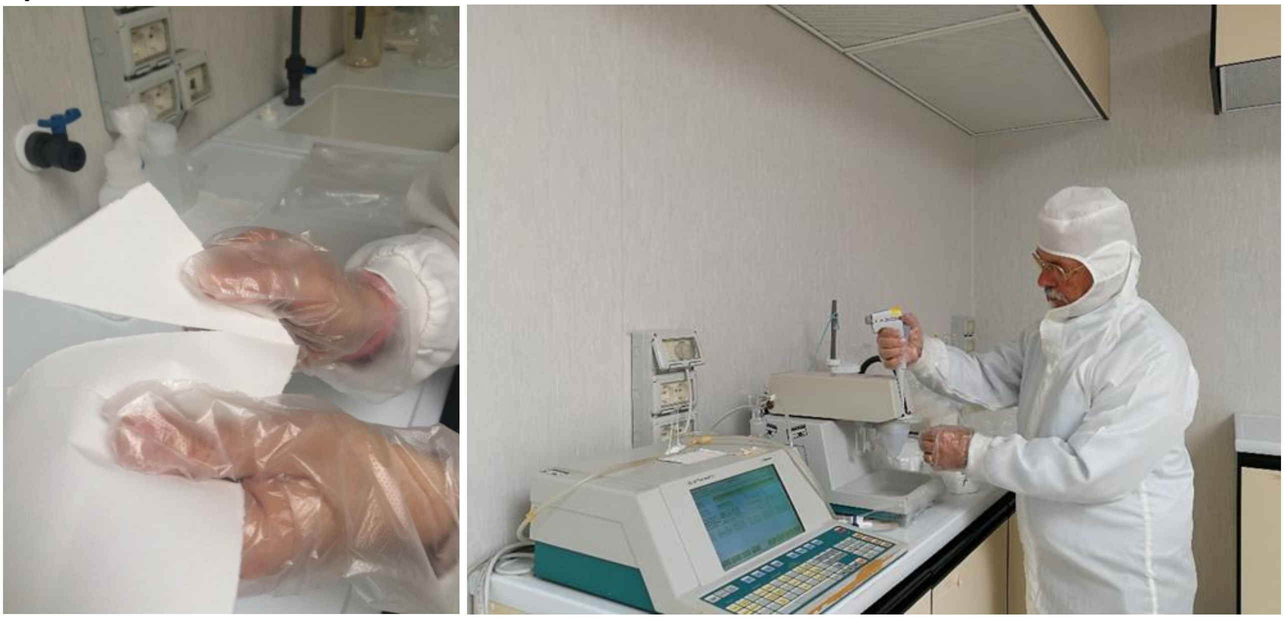

Figure S8. (**a**) Filter conditioning and weighing in the clean laboratory of Concordia Station, Antarctica. (**b**) Filter cutting and voltammetric analysis in our clean chemistry laboratory, Italy.

Section S8. Potential-time waveform applied in voltammetric analysis

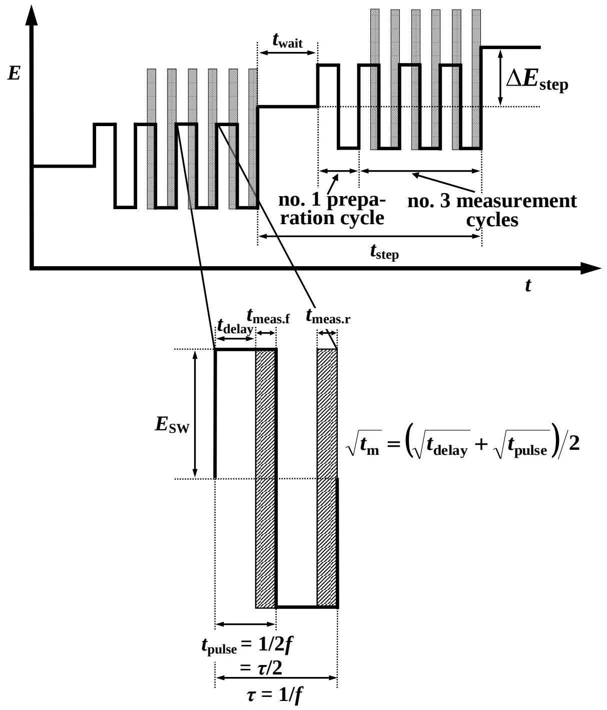

Figure S9. Potential-time waveform and current sampling scheme (dashed areas) in square-wave voltammetry. Symbol meanings: ΔE_{step}= step height in mV; t_{step}= step time in ms; t_{wait}= waiting time in ms, i.e. time elapsing between the beginning of the potential step and the start of the SW potential modulation; E_{sw}= SW amplitude in mV; t_{meas}= current sampling time, equal in forward ($t_{meas.f}$) and reverse ($t_{meas.r}$) pulses, in ms; f= frequency in Hz; t_{pulse}= pulse time in ms = 1/(2f); τ= period 1/f; t_m in ms, see text. Redrawn from [53] Illuminati et al., 2015.

Section S9. Original data

Table S8. Overall filter blanks (includes laboratory blanks).

Field blank filter	Cadmium blank		Lead blank		Copper blank	
	Concentration[a] ng l^{-1}	Mean ±SD ng l^{-1}	Concentration[a] ng l^{-1}	Mean ±SD ng l^{-1}	Concentration[a] µg l^{-1}	Mean ±SD µg l^{-1}
CS.1 (before)	10.3; 9.7; 10.1	10.0 ±0.3	90; 81; 85; 78	84 ±5	0.394; 0.390; 0.399; 0.357; 0.400	0.39 ±0.02
CS.2 (after)	9.6; 8.6; 10.5; 9.1	9.4 ±0.8	83; 89; 71; 86	82 ±8	0.358; 0.320; 0.300; 0.365	0.34 ±0.03
AT1.1 (before)	10.3; 10.9; 9.0	10.1 ±1.0	70; 83; 72	75 ±7	0.325; 0.330; 0.365	0.34 ±0.02
AT1.2 (after)	7.7; 11.2; 9.2	9.4 ±1.8	84; 86; 70	80 ±9	0.430; 0.393; 0.380	0.40 ±0.03
AT2.1 (before)	10.6; 10.5; 11.0	10.7 ±0.3	79; 81; 87	82 ±4	0.358; 0.322; 0.360; 0.330	0.34 ±0.02
AT2.1 (before)	11.4;10.5; 8.0	10.0 ±1.8	74; 65; 77; 86	76 ±9	0.412; 0.352; 0.360	0.37 ±0.03
Weighted mean ±SD		10 ±1		80 ±7		0.36 ±0.02

([a]) Conditions: 1/8 filter, 100 ml final solution after digestion.

Table S9. Original results from sample analyses.

Cadmium

Sample reference label	Gross sample concentration ([a]) ng l^{-1}	Gross sample mean ±SD ng l^{-1}	Net sample concentration ([b]) ng l^{-1}	Mass fraction µg g^{-1}	Atmospheric concentration pg m^{-3}
CS.1	86; 90; 98; 86	90 ±6	80 ±6	7.0 ±1.2	3.1 ±0.4
CS.2	21; 19; 17; 23	20 ±3	10 ±3	1.4 ±0.4	0.40 ±0.13
CS.3	59; 58; 53	56 ±3	46 ±3	5.2 ±0.5	2.0 ±0.2
AT1.1	24; 32; 25	27 ±4	17 ±4	6.6 ±1.6	0.70 ±0.18
AT1.2	15; 18; 14	16 ±2	6 ±2	1.3 ±0.4	0.25 ±0.09
AT1.3	12.1; 12.5; 11.9	12.2 ±0.3	2.2 ±1.0	1.0 ±0.5	0.09 ±0.04
AT2.1	46; 50; 54	50 ±4	40 ±4	8.4 ±1.0	0.98 ±0.14
At2.2	18; 15; 18	17 ±2	7 ±2	1.3 ±0.4	0.20 ±0.06

([a]) Conditions: 1/8 filter, 100 ml final solution after digestion. ([b]) Blank subtracted 10 ±1 ng l^{-1}.

Lead

Sample reference label	Gross sample concentration ([a]) µg l^{-1}	Sample mean ±SD µg l^{-1}	Net sample concentration ([b]) µg l^{-1}	Mass fraction mg g^{-1}	Atmospheric concentration pg m^{-3}
CS.1	1.64; 1.73; 1.68; 1.80; 1.55	1.68 ±0.09	1.60 ±0.09	0.14 ±0.02	62 ±7
CS.2	0.81; 0.86; 0.89; 0.88	0.86 ±0.04	0.78 ±0.04	0.11 ±0.01	31 ±3
CS.3	1.18; 1.03; 1.00	1.07 ±0.10	0.99 ±0.10	0.11 ±0.01	44 ±6
AT1.1	1.47; 1.34; 1.35; 1.14; 1.30; 1.21	1.30 ±0.12	1.22 ±0.12	0.47 ±0.06	50 ±7
AT1.2	0.669; 0.638; 0.640; 0.670	0.65 ±0.02	0.57 ±0.02	0.12 ±0.01	23 ±2
AT1.3	0.376; 0.372; 0.376	0.375 ±0.002	0.295 ±0.007	0.13 ±0.02	12 ±1
AT2.1	1.21; 1.07; 0.93; 1.02	1.06 ±0.12	0.98 ±0.12	0.20 ±0.03	24 ±4
At2.2	0.633; 0.542; 0.612	0.60 ±0.05	0.52 ±0.05	0.10 ±0.01	15 ±2

([a]) Conditions: 1/8 filter, 100 ml final solution after digestion. ([b]) Blank subtracted 0.080 ±0.007 µg l^{-1}.

Copper

Sample reference label	Gross sample concentration ([a]) µg l^{-1}	Gross sample mean ±SD µg l^{-1}	Net sample concentration ([b]) µg l^{-1}	Mass fraction mg g^{-1}	Atmospheric concentration ng m^{-3}
CS.1	58.7; 57.1; 68.1; 63.6; 66.1	63 ±5	63 ±5	5.4 ±0.9	2.4 ±0.3
CS.2	31.2; 24.4; 29.0	28 ±3	28 ±3	3.9 ±0.6	1.1 ±0.2
CS.3	8.6; 8.5; 8.6; 8.4	8.5 ±0.1	8.1 ±0.1	0.92 ±0.06	0.36 ±0.04
AT1.1	52.4; 46.5; 56.6; 52.5	52 ±4	52 ±4	20 ±2	2.1 ±0.3
AT1.2	5.7; 5.2; 4.7	5.2 ±0.5	4.8 ±0.5	1.06 ±0.14	0.20 ±0.03
AT1.3	3.79; 3.93; 3.58	3.77 ±0.17	3.41 ±0.17	1.53 ±0.12	0.14 ±0.02
AT2.1	5.8; 3.2; 4.8	4.6 ±1.3	4.2 ±1.3	0.88 ±0.28	0.10 ±0.03
At2.2	1.22; 1.31; 1.43	1.3 ±0.1	0.94 ±0.1	0.17 ±0.03	0.027 ±0.004

([a]) Conditions: 1/8 filter, 100 ml final solution after digestion. ([b]) Blank subtracted 0.36 ±0.02 µg l^{-1}.
